# AR71, Histamine H_3_ Receptor Ligand—In Vitro and In Vivo Evaluation (Anti-Inflammatory Activity, Metabolic Stability, Toxicity, and Analgesic Action)

**DOI:** 10.3390/ijms25158035

**Published:** 2024-07-23

**Authors:** Anna Stasiak, Ewelina Honkisz-Orzechowska, Zbigniew Gajda, Waldemar Wagner, Katarzyna Popiołek-Barczyk, Kamil J. Kuder, Gniewomir Latacz, Michał Juszczak, Katarzyna Woźniak, Tadeusz Karcz, Katarzyna Szczepańska, Marta Jóźwiak-Bębenista, Katarzyna Kieć-Kononowicz, Dorota Łażewska

**Affiliations:** 1 Department of Hormone Biochemistry, Faculty of Medicine, Medical University of Lodz, Żeligowskiego 7/9 Str., 90-752 Łódź, Poland; 2 Department of Technology and Biotechnology of Drugs, Faculty of Pharmacy, Jagiellonian University Medical College in Kraków, Medyczna 9 Str., 30-688 Kraków, Poland; 3 Laboratory of Cellular Immunology, Institute of Medical Biology of Polish Academy of Sciences, 106 Lodowa Str., 93-232 Łódź, Poland; 4 Department of Neurochemistry, Maj Institute of Pharmacology, Polish Academy of Sciences, Smętna 12 Str., 31-343 Kraków, Poland; 5 Department of Molecular Genetics, Faculty of Biology and Environmental Protection, University of Lodz, Pomorska 141/143 Str., 90-236 Łódź, Poland; 6 Department of Medicinal Chemistry, Maj Institute of Pharmacology, Polish Academy of Sciences, Smętna 12 Str., 31-343 Kraków, Poland; 7 Department of Pharmacology and Toxicology, Medical University of Lodz, Żeligowskiego 7/9 Str., 90-752 Łódź, Poland

**Keywords:** histamine H_3_ receptor, chalcone, metabolic stability, cytotoxicity, neuroprotection, analgesic activity

## Abstract

The future of therapy for neurodegenerative diseases (NDs) relies on new strategies targeting multiple pharmacological pathways. Our research led to obtaining the compound AR71 [(E)-3-(3,4,5-trimethoxyphenyl)-1-(4-(3-(piperidin-1-yl)propoxy)phenyl)prop-2-en-1-one], which has high affinity for human H_3_R (K*_i_* = 24 nM) and selectivity towards histamine H_1_ and H_4_ receptors (K*_i_* > 2500 nM), and showed anti-inflammatory activity in a model of lipopolysaccharide-induced inflammation in BV-2 cells. The presented tests confirmed its antagonist/inverse agonist activity profile and good metabolic stability while docking studies showed the binding mode to histamine H_1_, H_3_, and H_4_ receptors. In in vitro tests, cytotoxicity was evaluated at three cell lines (neuroblastoma, astrocytes, and human peripheral blood mononuclear cells), and a neuroprotective effect was observed in rotenone-induced toxicity. In vivo experiments in a mouse neuropathic pain model demonstrated the highest analgesic effects of AR71 at the dose of 20 mg/kg body weight. Additionally, AR71 showed antiproliferative activity in higher concentrations. These findings suggest the need for further evaluation of AR71’s therapeutic potential in treating ND and CNS cancer using animal experimental models.

## 1. Introduction

Despite advancements in medicine, the treatment of neurodegenerative diseases (NDs) remains a significant challenge. The exact cause of neuronal death is not fully understood for many of these diseases, making treatment primarily symptomatic [1,2,3,4,5]. On the one hand, the classification of NDs is based on their different causes, symptoms, and specific morphological and neurochemical pathological changes. On the other hand, common pathogenetic mechanisms have been observed in the course of these diseases and the associated changes in neuron morphology and function, linking, among others, diseases like Alzheimer’s disease (AD) and Parkinson’s disease (PD), two of the most common NDs [2,4,6]. It is a well-established fact that AD and PD are progressive, irreversible, and have a multifaceted pathogenesis. The involvement of various processes that react to an unknown primary etiological factor is considered [2,3]. The probable causes include genetic predispositions and environmental factors associated with exposure to toxic substances [1,2,3]. It is crucial to note that only age and genetic conditions are considered non-modifiable risk factors, with the latter affecting only 10–20% of patients (the remaining cases are treated as idiopathic). Pathogenetic factors comprise an excessive aggregation of proteins in neurons and in the extraneuronal space, oxidative stress, mitochondrial damage, excitotoxicity related to the overactivity of the glutamatergic system, immunological and inflammatory processes (both in the CNS and the periphery), disruption of the gut-brain axis (the influence of the microbiome on the penetration of harmful substances into the brain), activation of microglia, metal dyshomeostasis, and, of course, deficits of neurotransmitters in the brain, particularly acetylcholine (ACh) in AD and dopamine (DA) in PD [1,2,3,4,5,6,7]. It is important to note that we do not fully understand which of these mechanisms is crucial and how they interact with each other. These potential causes and mechanisms are the main focus of current treatment approaches. Regrettably, despite years of extensive research, we still lack completely effective pharmacotherapy.

AD is marked by memory deficit and a significant decline in other cognitive functions. Based on epidemiological data, there are currently over 55 million people worldwide suffering from AD [3]. According to the WHO Global Status Report 2021 [8], it is estimated that by 2030, this number will increase to 78 million; in 25 years, it will reach 139 million. AD pathophysiology involves the buildup of amyloid-β (Aβ), which leads to the formation of senile plaques and neurofibrillary tangles inside cells due to hyperphosphorylated tau protein [1,4,5]. Basic AD therapy is based on the use of acetylcholinesterase (AChE) inhibitors (donepezil, galantamine, rivastigmine) and N-methyl-D-aspartate (NMDA) receptor antagonist (memantine) [4,5]. In 2021, almost 20 years after memantine was introduced, the U.S. Food and Drug Administration (FDA) approved a new drug called Aducanumab. Aducanumab is a monoclonal antibody that targets soluble Aβ plaques and has been reported to affect two main pathophysiologic hallmarks of AD (Aβ and tau) [9]. In January 2023, another antibody, Lecanemab, a humanised immunoglobulin gamma 1 (IgG1) against aggregated soluble and insoluble forms of Aβ peptide, received a positive FDA opinion [10]. 

PD is the second most common neurodegenerative disease (after AD); it affects 2–3% of the world’s population over 65 years of age. The incidence is 5–35 new cases per 100,000. people per year [11]. PD is associated with the accumulation of aggregated α-synuclein (Lewy bodies) and neurodegeneration of the substantia nigra, which leads to decreased DA levels in the brain, causing motor symptoms [2,7]. The treatment for PD involves various symptomatic pharmacological strategies aimed at increasing DA levels [5]. The gold standard treatment is oral levodopa (DA precursor), administered with an aromatic amino acid decarboxylase inhibitor. Other medications used to treat PD are DA receptor agonists (pramipexole, ropinirole, rotigotine, piribedil), amantadine (NMDA receptor antagonist), monoamine oxidase B (MAO B) and catechol-*O*-methyltransferase (COMT) inhibitors (selegiline, rasagiline, safinamide and entacapone, tolcapone, opicapone), and anticholinergics (biperiden, pridinol). In addition to oral drugs, treatment options also include subcutaneous injections of apomorphine (DA agonist with high affinity to dopamine D_1_, D_2_, and D_3_ receptors) in the form of repeated or continuous injection, as well as infusions of phoslevodopa/phoscarbidopa and enteral administration of levodopa/carbidopa (Duodopa). There is also a focus on deep brain stimulation, improving infusion therapies, personalising levodopa treatment (Flexilev microtablets), gene therapy, and neuroprotection [5,12]. 

In the course of both diseases, attention is also paid to stabilising the functions of other neurotransmission systems. The future of AD and PD pharmacotherapy involves, among others, multitarget-directed ligands (MTDLs), according to the “one drug—many targets” paradigm [13]. Numerous experimental studies indicate that MTDLs improve both effectiveness and therapeutic safety compared with drugs that are based on only one neurochemical mechanism [13,14,15,16,17]. 

As previously described in the literature (by us and other authors), histamine H_3_ receptor (H_3_R) antagonists seem to be an attractive pharmacological target of MTDLs because of their location and functions [17,18]. The H_3_Rs are G protein-coupled receptors (GPCRs) predominantly expressed in the CNS. They act as auto- and heteroreceptors and regulate the level of histamine and other neurotransmitters (such as ACh, DA, serotonin, noradrenaline, GABA, and glutamate) [19,20,21]. In this way, H_3_Rs in the CNS play a role in diverse physiological and pathological mechanisms. 

Examples include agents that combine the activity of MAO-B blocker and the H_3_R antagonism, as well as compounds that simultaneously inhibit the activity of H_3_R and AChE. These MTDLs are aimed at treating PD and AD, respectively [14,17,18,22,23,24,25,26]. 

Research has demonstrated that H_3_Rs are widely distributed not only in neurons but also in microglia, where they regulate the inflammatory response [27]. It is also well-documented that histamine H_3_R antagonists have pain-relieving effects in various experimental animal pain models [28,29]. Additionally, studies have revealed the presence of H_3_Rs on various cancer cells and how tumour growth can be influenced by H_3_R antagonists [30,31,32]. 

While looking for an effective therapy for NDs, we focused on the pleiotropic effect of chalcones (flavonoid precursors). These compounds have long been known to have broad biological activity [33] and have anti-inflammatory, antimitotic, and immunosuppressive effects [34,35]. Research proves their therapeutic effectiveness in the course of bacterial, viral, fungal, and protozoal diseases. Attention is also given to their anti-cancer activity [36,37]. Chalcones are also reported to have neurotrophic effects [38] and are suggested to be effective in NDs [39,40]. 

**Figure 1:**
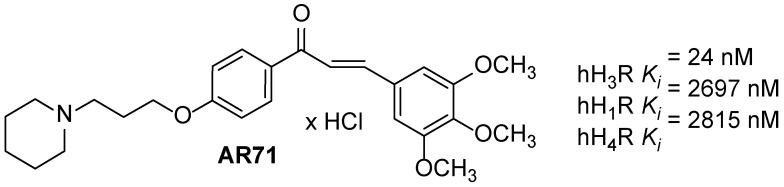
Structure and histamine receptors affinities of AR71.

Our studies led to obtaining the compound AR71 [(E)-3-(3,4,5-trimethoxyphenyl)-1-(4-(3-(piperidin-1-yl)propoxy)phenyl)prop-2-en-1-one], which has high affinity for human H_3_R (K*_i_* = 24 nM) and selectivity towards histamine H_1_ and H_4_ receptors (K*_i_* > 2500 nM). The structure of AR71 combines a piperidinylpropoxy motif typical for H_3_R antagonists/inverse agonists and a chalcone moiety (Figure 1). Preliminary studies confirmed its positive anti-inflammatory activity in a model of LPS-induced inflammation in BV2 cells [41]. 

The current research was built upon our previous results [40]. The objectives were to assess AR71’s metabolic stability, conduct docking studies for histamine H_1_, H_3_, and H_4_ receptors, and enhance our knowledge of its cytotoxicity and pharmacological activity, including anti-inflammatory and neuroprotective properties. This involved in vitro tests with various cell lines and in vivo experiments using a mouse neuropathic pain model.

## 2. Results and Discussion

### 2.1. Intrinsic Activity Toward H_3_R—Assay in HEK Cells Expressing Human Recombinant H_3_R

The intrinsic activity of AR71 was evaluated in a 3′,5′-cyclic adenosine monophosphate (cAMP) accumulation assay in human embryonic kidney (HEK) 293 cells, stably expressing the hH_3_R [42]. AR71 can be classified as an H_3_R antagonist/inverse agonist as it was able to counteract inhibition of cAMP production by (R)-(-)-α-methyl histamine in forskolin-stimulated cells. The IC_50_ value obtained in this assay is 83.5 ± 11.9 nM (Figure S1, Supplementary Materials), and it is in accordance with its K_i_ value at hH_3_R (K_i_ = 24 nM).

### 2.2. In Silico Docking Studies

#### 2.2.1. Docking Studies to Histamine H_3_ Receptor

The recent publication of H_3_R complexed with an antagonist (PDB ID: 7F61 [43]) allowed for confirmation of putative ligand–receptor interactions and a better understanding of the receptor function itself. For the purpose of molecular docking studies, AR71 was chosen. The compound was characterised by a relatively high calculated MMGBSA dG bind value of −124.52 kcal/mol. It occupied the H_3_R binding pocket in a similar mode preserving a crucial histamine H_3_R antagonist/inverse agonist interaction, namely, salt bridge and/or hydrogen bond formation between protonated amine nitrogen and D114^3.32^ (superscripts denote Ballesteros–Weinstein numbering) [44]. Additional stabilisation through cation–π interactions with caging Y115^3.33^ and F398^7.39^ was also found. The latter was the π-stacking proximal aromatic ring, followed by a H-bond between Y91^2.61^ and carbonyl group oxygen. The distal, tri-substituted aromatic ring was directed towards the extracellular space and was stabilised by H187(EL2).

**Figure 2:**
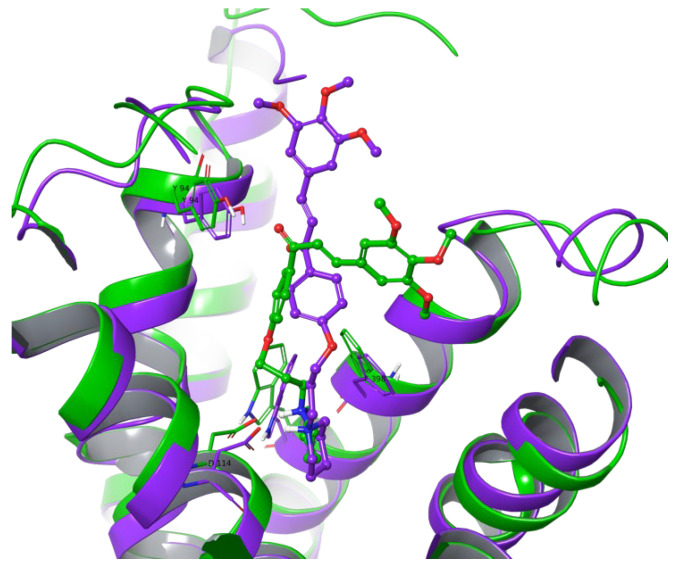
Histamine H_3_ receptor—comparison of the 1st (violet) and 1000th (green) frame for the recorded 7F61-AR71 complex. The upper parts of TM4 and TM5, as well as EL2, were removed for better viewing clarity.

**Figure 3:**
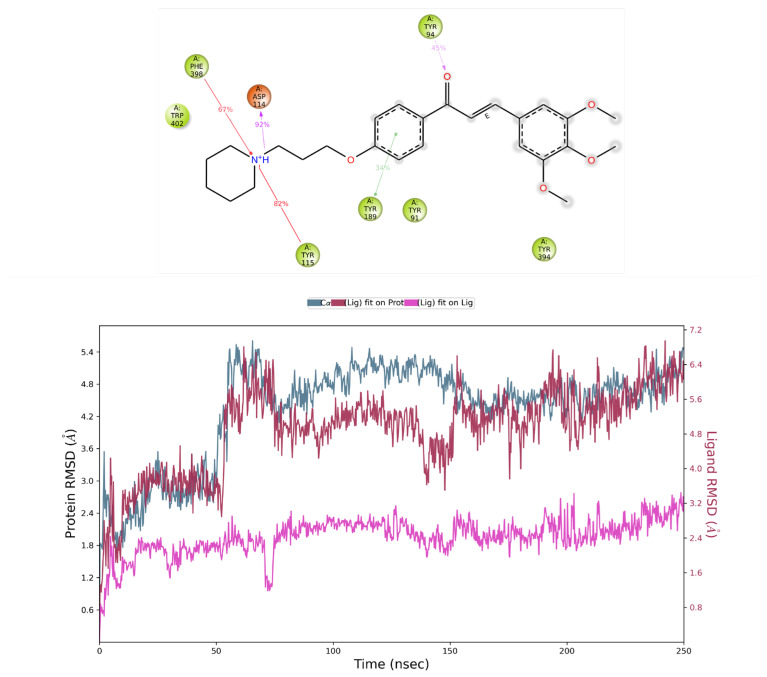
Results for histamine H_3_ receptor. Top left panel: AR71 contact summary through the recorded simulation time. Lower panel: RMSD evolution of a protein (left Y-axis) and AR71 (right Y-axis; pink—fit on ligand, maroon—fit on protein). The protein–ligand contact timeline can be found in the Supplementary Materials (Figure S2).

The stability of the calculated pose was further evaluated by means of, firstly, 100 ns molecular dynamics (MD) simulations. Because of a slight increase in the observed ligand RMSD value in the last 10 ns of simulation, we decided to perform a longer 250 ns simulation to check or confirm the stability of the ligand in the binding site. From the simulation, 20 frames were selected (starting pose and after each 12.5 ns). In fact, AR71 was stable only at the beginning of the simulation (up until ca. 50 ns), and from then on, because of a highly flexible alkyl linker between protonated piperidine and the proximal aromatic moiety, this part of the molecule wandered between ECL2 and ECL3 in such a way that at the end of the simulation, the ligand had evolved to a conformation where the distal trimethoxyphenyl ring was facing TM6/TM7 and mainly stabilised with π-π interactions with aromatic EL2 residues. Despite the mirror-image conformation (Figure 2), the key interaction with D114^3.32^ was retained through 92% of the simulation time, and additional interactions with Y115^3.33^ and F398^7.39^ were retained for 82% and 67% of the recorded trajectory time, respectively. Also, the carbonyl moiety switched the interacting partner to Y94^2.64^ (Figure 3). The average binding free energy of the complex was calculated for the recorded trajectory and resulted in MMGBSA dG bind = −82.01 kcal/mol—a significantly lower value than that of the starting complex (−124.52 kcal/mol). Moreover, paying attention to per-residue RMSD, binding site residues were relatively stable during the whole simulation, with values in the range of 1–1.4 (Table S1, Supplementary Materials), suggesting that the ligand might stabilise the inactive state of the receptor. Further, the postulated H_3_R inactive state 3–7 lock between D114^3.32^ and W402^7.43^ [45] was observed through the whole recorded trajectory, with short breakage between 550th and 650th, which may give additional confirmation of the presumed stabilisation of the inactive state of the receptor by the tested AR71 ligand.

#### 2.2.2. Docking Studies to Histamine H_1_ and H_4_ Receptors

Molecular docking for AR71 was carried out using the histamine H_1_ receptor (H_1_R) structure complexed with antagonist doxepin (PDB ID: 3RZE [46]) and histamine H_4_ receptor (H_4_R) complexed with native ligand histamine (PDB ID: 7YFC [47]). The binding mode to H_1_R was characterised by a hydrogen bond between D178(EL2) and the protonated amine nitrogen, as well as the π-stacking interaction between the trimethoxy phenyl moiety and F432^6.52^ (Ballesteros–Weinstein notation in superscripts, according to GPCRdb [48]). The calculated MMGBSA dG bind value amounted to −4.69 kcal/mol. The binding mode to H_4_R was characterised by a salt bridge between the protonated amine and E182^5.46^, a hydrogen bond between the central ether oxygen atom and Q347^7.42^, and the π-stacking interaction between the trimethoxy phenyl moiety and PHE344^7.39^, with a calculated MMGBSA dG bind value of −31.64 kcal/mol. In comparison with native ligands, only the salt bridge interaction was preserved.

Additional induced-fit docking to H_1_R showed an improved but not sufficient binding mode. In most cases, AR71 appeared as an upside-down cluster (protonated piperidine facing the extracellular space), retaining the previously mentioned interactions. In two cases, however, a pose for which salt bridge formation between the protonated amine and D107^3.32^, supported by π–cation interactions between K179(EL2) and π-stacking with H450^7.35^, was found. The calculated MMGBSA dG bind value amounted to a slightly higher value of −72.32 kcal/mol. Induced-fit docking also showed improvement in binding to H_4_R—namely, by means of the calculated salt bridge between the protonated amine and E182^5.46^, the hydrogen bond between the oxygen atom of the trimethoxy phenyl moiety and Y72^2.61^, the π-stacking interaction between the trimethoxy phenyl moiety and F344^7.39^, and the π-stacking interaction between the medial phenyl moiety and Y95^3.33^. Both of the latter have already been described in the literature [49,50]. The calculated MMGBSA dG bind value amounted to −104.75 kcal/mol. Overall, the calculated binding poses of AR71 in the respective crystal structures present low putative binding affinity to the H_1_R and H_4_R receptors, which aligns with the results of biological tests.

**Figure 4:**
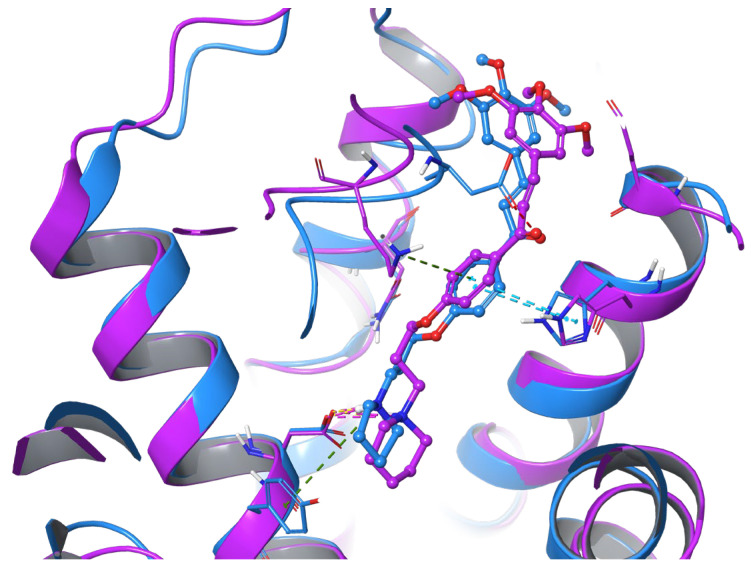
Superimposition of the first (blue; t = 0 ns) and last (violet; t = 100 ns) frames for the 3RZE—AR71 complex (H_1_R). Dashed lines denote molecular interactions: H-bond (yellow), salt bridge (pink), π-π (blue), cation-π (green).

**Figure 5:**
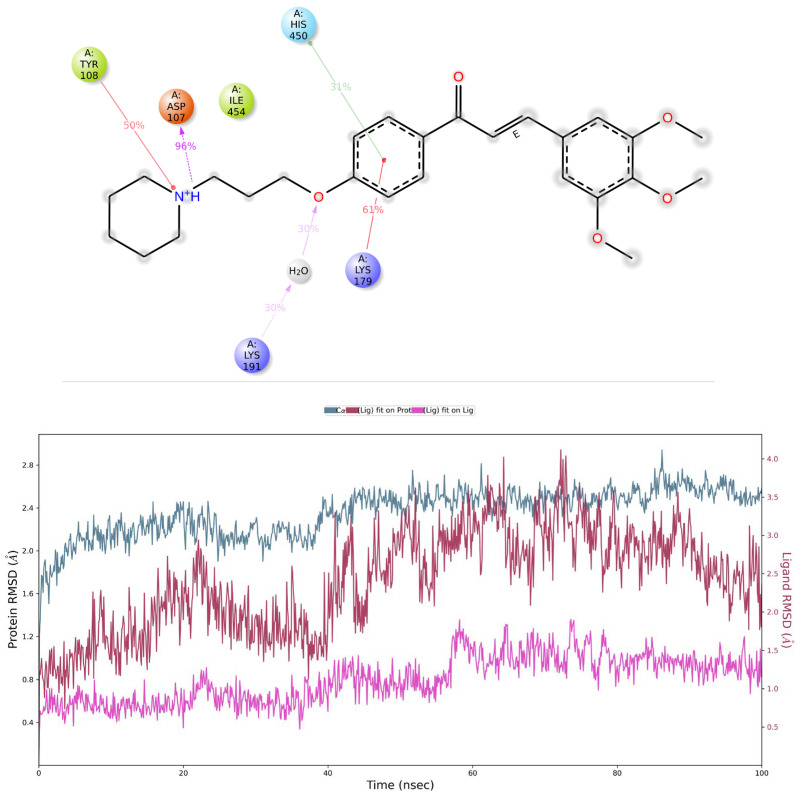
Results for histamine H_1_ receptor. Top panel: protein–ligand contact summary through the recorded simulation time. Lower panel: RMSD chart of a protein (left Y-axis; blue) and ligand (right Y-axis; pink—fit on ligand, maroon—fit on protein). The protein–ligand contact timeline can be found in the Supplementary Materials (Figure S3).

To investigate the stability of calculated binding poses further, an additional evaluation by means of molecular dynamics simulation was performed (duration 100 ns, 1000 frames, 100 ps each). The starting pose and 20 more frames were selected, each after 5 ns (Figure 4). In both cases, except for the stable salt bridge between the protonated amine and key interacting residues D107^3.32^ and E182^5.46^ (retained through 96% and 87% of the simulation time, respectively), for each frame, AR71 showed much instability, owed to flexible linker chain, even with additional stabilisation through either the π–cation interaction between the central phenyl and K179(EL2) (H_1_R), retained through 61% of the simulation time, or H-bonds between the distal methoxy group and R341^7.36^ (H_4_R). The RMSD value fluctuated according to shifts in the ligand’s position. The calculated MMGBSA dG bind values for the first and last frames decreased through the trajectory time, from −72.32 kcal/mol to −63.05 kcal/mol and from −104.75 to −73.05 for the H_1_R and H_4_R complexes, respectively, further explaining poor binding affinity. An exemplary comparison of the first and last collected frames for the H_1_R-AR71 complex and corresponding simulation interaction diagrams are presented in Figure 4 and Figure 5, respectively.

### 2.3. Metabolic Stability of AR71

The metabolic stability of AR71 was assessed in vitro using human liver microsomes (HLMs). AR71 was rather metabolically stable. After 120 min of incubation with HLMs, 85% of AR71 remained unchanged. However, during this time, six metabolites were formed (Figure S4a–d, Supplementary Materials). The metabolite M1 was formed in the highest amount (5.5%). The others were formed in amounts of 0.6% to 1.2%. Based on an analysis of their molar masses, and with the help of MetaSite 6.0.1, a type of metabolic transformation was proposed. The identified in vitro AR71 metabolic pathways and the most probable site of metabolism predicted by MetaSite 6.0.1 are shown in Table 1.

### 2.4. Assessment of AR71 Cytotoxicity

The cytotoxicity of AR71 was tested using two human physiological cell lines, including astrocytes and peripheral blood mononuclear cells, and one cancer line—neuroblastoma cells. Additionally, in studies on neuroblastoma, the effect of the compound on the cell cycle was checked by determining the 2N/4N DNA ratio index (High Content Analysis Image Cytometry).

#### 2.4.1. Effect of AR71 on the Viability of the Human Astrocyte Cell Line 

**Figure 6:**
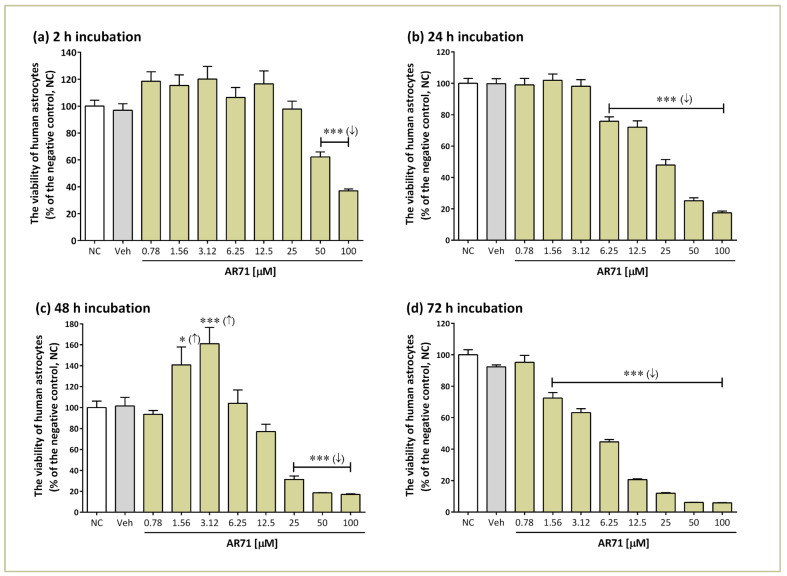
Effect of AR71 on the viability of human astrocytes evaluated using the MTT assay. The viability for individual samples was calculated relative to the negative control (NC, untreated cells) after 2 h (**a**), 24 h (**b**), 48 h (**c**), and 72 h (**d**) of incubation with AR71. The positive control was astrocytes incubated with 0.1% DMSO (Veh). Bars represent means with SEMs of six independent experiments and are expressed as a percentage of untreated control cells. One-way ANOVA and Dunnett’s multiple comparisons test: * *p* < 0.05, *** *p* < 0.001 vs. the negative control (NC); (↑) - increase; (↓) - decrease.

We investigated the effect of AR71 in a wide range of concentrations (0.78–100 μM) on the viability of human astrocyte cell lines after 2, 24, 48, and 72 h of incubation (Figure 6). Prolonged incubation of human astrocytes with AR71 caused a dose-dependent decrease in cell viability. After 2 h of incubation, cytotoxicity of AR71 was observed only at the highest concentrations of 50 and 100 μM (IC_50_ = 41.28 μM). With longer incubation times, cytotoxicity increased; specifically, after 24 h, the IC_50_ was 20.21 μM, and after 48 and 72 h, it was 12.53 and 9.69 μM, respectively (Table 2; middle column). After 48 h, statistically significant proliferation was observed at concentrations of 1.56 and 3.12 μM, but this effect did not persist after 72 h. DMSO (a solvent for AR71) did not significantly impact cell viability.

#### 2.4.2. Effect of AR71 on Human Peripheral Blood Mononuclear Cell Viability (PBMCs)

**Figure 7:**
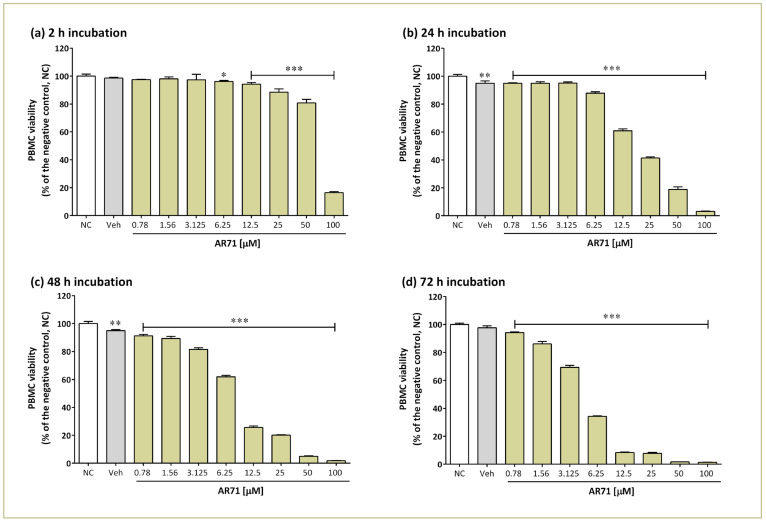
Effect of AR71 on PBMC viability. The viability of individual samples was calculated relative to the negative control (NC, untreated cells) after 2 h (**a**), 24 h (**b**), 48 h (**c**), and 72 h (**d**) of incubation with AR71. The positive control was PBMCs incubated with 0.1% DMSO (Veh). Bars represent the means and SEMs of six independent experiments and are expressed as a percentage of untreated control cells. One-way ANOVA and Dunnett’s multiple comparisons test: * *p* < 0.05, ** *p* < 0.01, *** *p* < 0.001 vs. the negative control (NC).

In the next step, we investigated the effect of AR71 on the viability of human PBMCs. The experiment was conducted with the same compound doses and incubation times as the astrocyte studies. The results are presented in Figure 7 and Table 2 (left column).

Generally, the cytotoxic effect of AR71 against PBMCs was directly proportional to the concentration of the compound and the incubation time. According to the obtained data, the 2 h incubation of AR71 at concentrations of 0.78, 1.56, and 3.125 µM did not cause a cytotoxic effect (Figure 7a). In the case of higher concentrations (6.25–100 µM), a statistically significant decrease in the number of PBMCs compared with the negative control was noted (one-way ANOVA and Dunnett’s multiple comparisons test, *p* < 0.001), but for concentrations in the range of 6.25–50 µM, it did not exceed 20% (Figure 7a). 

With a longer incubation (24 h, 48 h, and 72 h), each AR71 concentration used resulted in a statistically significant decline in the viability of PBMCs compared with the control (one-way ANOVA and Dunnett’s multiple comparisons test, *p* < 0.001). However, a decrease in abundance of around 50% was only caused by higher compound concentrations. It is worth noting that the highest concentration of AR71 used (100 µM) showed high toxicity, regardless of the incubation time, causing a decrease in the number of PBMCs by 80–98% (Figure 7a–d). 

In summary, the resazurin reduction assay on PBMCs demonstrated AR71 toxicity in the micromolar range, as documented by the calculated IC_50_ values (Table 2).

#### 2.4.3. Effect of AR71 on the Viability of the Human Neuroblastoma Cell Line (SH-SY5Y)

**Figure 8:**
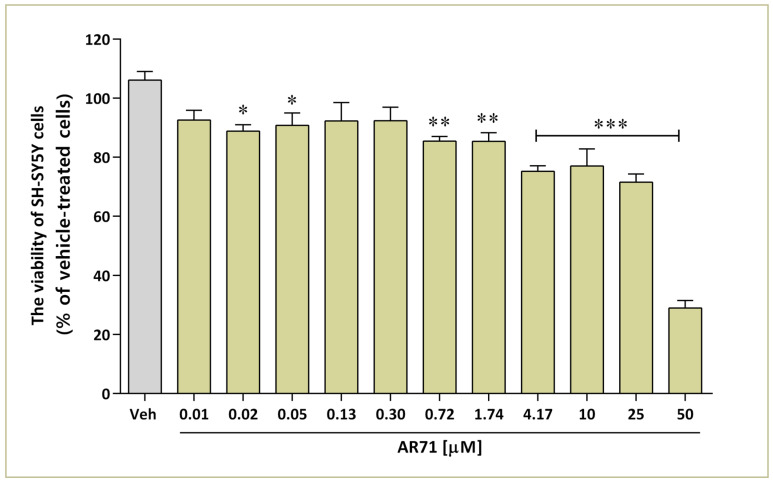
Effect of AR71 on the viability of neuroblastoma (SH-SY5Y) cells after a 24 h incubation. The viability of individual samples was calculated relative to the vehicle-treated cells (0.1% DMSO). Bars represent the means and SEMs of two independent experiments, each consisting of four replicates per treatment group. One-way ANOVA with post hoc Dunnett’s test: * *p* < 0.05; ** *p* < 0.01; *** *p* < 0.001 vs. Veh (vehicle-treated cells).

The toxicity of AR71 in neuroblastoma cells was evaluated with the 3-(4,5-dimethylthiazol-2-yl)-5-(3-carboxymethoxyphenyl)-2-(4-sulfophenyl)-2H-tetrazolium (MTS) assay. The cells were cultured with AR71 at various concentrations ranging from nanomolar to micromolar for 24 h. The results showed that AR71 in concentrations up to 0.72 μM did not affect cell viability (Figure 8). This study revealed a significant decrease at *p* < 0.001 in cell metabolic activity starting from a concentration of 4.17 μM. The most substantial loss in neuroblastoma viability, which amounted to 73% compared with the control cells, was observed at a concentration of 50 μM. The IC_50_ value, determined from the MTS assay data, was 24.93 μM (Figure 8). 

#### 2.4.4. Evaluation of the Growth Rate of Neuroblastoma Cells in the Presence of AR71 with High Content Analysis Image Cytometry

DNA cell content analysis and proliferation assays were performed to investigate the cytotropic effects of AR71 on immature nerve cells, the SH-SY5Y cell line. Although other H_3_R antagonists (e.g., ciproxifan) have been shown to suppress the proliferation and metastatic potential of cancer cells [32], the examined neuroblastoma cells were reported to express H_3_R negligibly [51].

**Figure 9:**
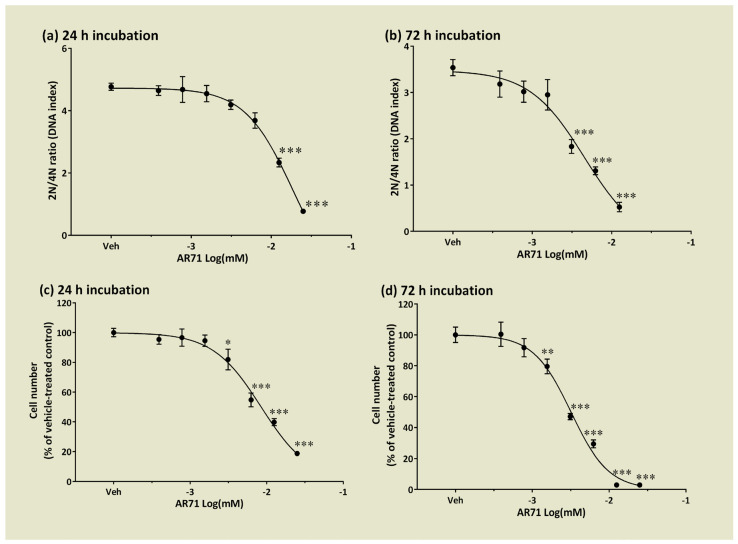
The effects of AR71 on the growth rate of neuroblastoma SH-SY5Y cells. H-SY5Y cells were treated with either vehicle (0.1% DMSO) or increasing concentrations of AR71 for 24 and 72 h. (**a**,**b**) Cellular DNA content was determined using image cytometry, based on Hoechst-33342 fluorescence intensity of single cell nuclei. The analysis was conducted using the Cell Cycle Bioapplication V3, and the results are presented as a DNA 2N/4N ratio. This involved analysing the nuclei fluorescence of 500 cells in each of 4 replicates. (**c**,**d**) Cell numbers were determined using image cytometry. They were calculated as the sum of Hoechst-33342-stained cell nuclei counted in 16 microscopic fields per well (4 replicates per treatment) and are presented as a percentage of the vehicle-treated control (Veh). Each value represents the mean ± SEM from at least three independent experiments. One-way ANOVA and Dunnett’s multiple comparisons test: * *p* < 0.05, ** *p* < 0.01 or *** *p* < 0.001 vs. control counterparts.

To evaluate the growth dynamics of SH-SY5Y cells in the presence of AR71, neuroblastoma cells were treated with increasing concentrations (0.39–25 µM) of the compound tested for 24 or 72 h. Using image cytometry, we calculated the DNA content index as a 2N/4N DNA ratio to analyse the cell cycle distribution after 24 and 72 h of incubation with AR71. Figure 9 shows that incubation with 3.125–25 µM of AR71 resulted in cell cycle arrest at the G2/M phase (4N) as the 2N/4N DNA ratio significantly decreased compared with the vehicle-treated cells. The IC_50_ values were 18.8 µM and 4.54 µM after 24 and 72 h of incubation with the compound tested, respectively. The inhibitory effect of AR71 on cell cycle progression directly resulted in a reduced number of neuroblastoma cells, as observed in complementary experiments. AR71 inhibited SH-SY5Y cell growth in 24 to 72 h, with IC_50_ values of 8.46 µM and 3.19 µM, respectively.

Surprisingly, we observed a slight discrepancy in the side effects of AR71 on metabolic viability (mitochondrial activity) and cell cycle/proliferation in neuroblastoma cells (24.93 μM vs. 8.46 μM at 24 h). According to recent reports [52], polyphenols and many anti-cancer drugs acting through the generation of intracellular ROS and induction of cell cycle arrest may stimulate mitochondrial biogenesis and/or increase mitochondrial mass. Thus, testing cell viability with MTT/MTS tests based on mitochondrial activity may result in an underestimation of drug-induced cell killing compared with cell number counting. Knowing the limitations of MTT/MTS, we employed complementary tests, i.e., proliferation and cell cycle experiments, to support and consolidate the obtained results and draw conclusions from multiparameter cell viability experiments. 

Our cytotoxicity studies on human PMBCs, astrocytes, and neuroblastoma cells demonstrated AR71 toxicity in the micromolar range, as documented by the calculated IC_50_ parameters (Table 2, Figure 7 and Figure 8). The IC_50_ values for the 24 h incubation of all three tested cell lines (two physiological and one cancer) with AR71 were very similar.

The compound’s antiproliferative effect in higher doses and its well-documented anti-inflammatory activity predispose AR71 to tests for anticancer activity. 

So far, the modulatory effect on cancer processes has been separately demonstrated for H_3_Rs [30,31,32] and chalcones [36,37]. The antiproliferative effect of AR71, a hybrid compound combining the properties of an H_3_R antagonist and chalcone moiety, confirms these effects. 

### 2.5. Assessment of the Neuroprotective Potential of AR71

The AR71 compound has been studied for its potential neuroprotective activity in rotenone-induced in vitro toxicity. Rotenone is a naturally occurring neurotoxin found in the seeds and stems of several plants, such as the jicama vine, and in the roots of several other members of the *Fabaceae* family [53,54]. It is used as an ingredient in insecticides and herbicides. The compound is a mitochondrial toxin that is a potent, reversible, and competitive inhibitor of complex I (NADH-CoQ reductase) in the respiratory chain [53,55]. As previously mentioned, PD is an ND with an unknown aetiology. Possible causes include oxidative stress, mitochondrial dysfunction, and neuroinflammation [3,7]. According to epidemiological data, there are more PD incidences in regions where rotenone plant protection products are widely used [56,57,58]. Moreover, either intracerebral or peripheral, rotenone administration is a method used to induce PD in experimental conditions. This model reflects many of the key features of the disease including progressive death of dopaminergic neurons and formation of α-synuclein inclusions. The toxin also destabilises microtubules [53,55]. 

**Figure 10:**
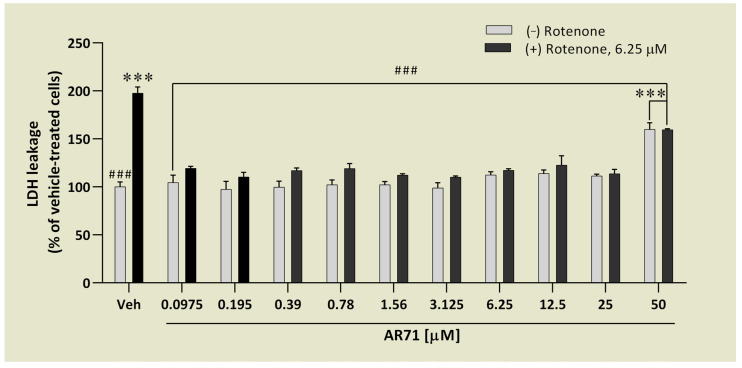
LDH leakage in SH-SY5Y cells treated with AR71 in the absence or presence of rotenone. (−) rotenone: SH-SY5Y cells were treated with increasing concentrations of AR71 (0.0975–50 μM). (+) rotenone 6.25 μM: SH-SY5Y cells were pre-treated with increasing concentrations of AR71 (0.0975–50 μM) for 1 h before stimulation with rotenone (6.25 μM) for another 24 h. Each point represents the mean ± SEM of two independent experiments, each consisting of four replicates per treatment group. Data are presented as a percentage of control cells treated with 0.1% DMSO (Veh). Statistical analysis by one-way ANOVA with post hoc Dunnett’s test: *** *p* < 0.001 vs. Veh; ^###^ *p* < 0.001 vs. rotenone-treated cells.

The LDH assay, in which the integrity of the cell membrane is tested, showed that only the highest concentration of 50 μM caused LDH leakage by almost 60% compared with the control cells (Figure 10). The rest of the concentrations (0.0975–25 μM) did not disturb the cell membrane. To test neuroprotective activity, SH-SY5Y cells were pre-treated for 1 h with various concentrations of AR71, after which rotenone (6.25 μM) was added and the cells were cultured for another 24 h. We found that SH-SY5Y cells treated with rotenone alone characterised twice as much LDH leakage as control cells. Co-treatment with AR71 protected cells from the toxic effect of rotenone, resulting in LDH leakage ranging from 10 to 22% only. 

**Figure 11:**
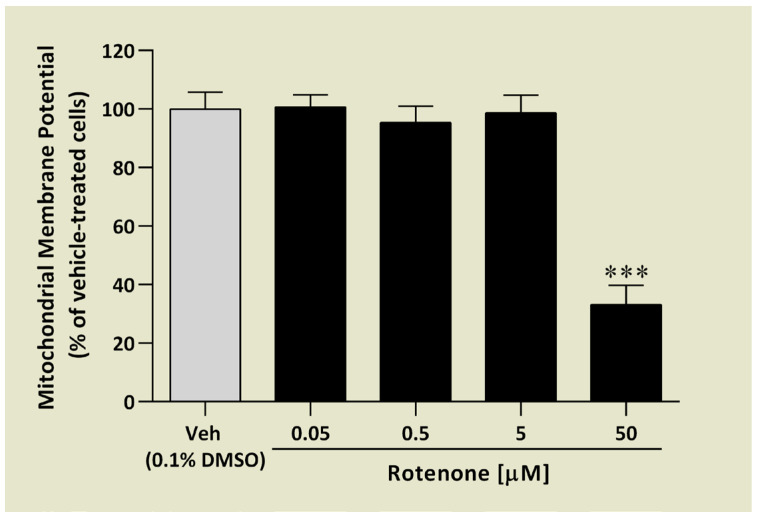
Dose–response effect of rotenone on mitochondrial membrane potential in SH-SY5Y cells after 3 h. Each point represents the mean ± SEM of two independent experiments, each consisting of four replicates per treatment group. Data are presented as a percentage of control cells treated with vehicle (Veh, 0.1% DMSO). One-way ANOVA with post hoc multiple comparison Dunnett’s test: *** *p* < 0.001 vs. Veh.

Neurotoxin-like rotenone induces oxidative stress inside cells that might influence mitochondrial membrane potential (MMP). Maintaining unaffected MMP is essential for neuroprotection [59]. In our study, we tested the effect of AR71 on MMP in the presence and absence of rotenone. First, we optimised the concentration of rotenone, which significantly reduced the level of MMP. Figure 11 shows that 50 μM of rotenone reduced MMP by 67% compared with the control cells after 3 h of stimulation. 

**Figure 12:**
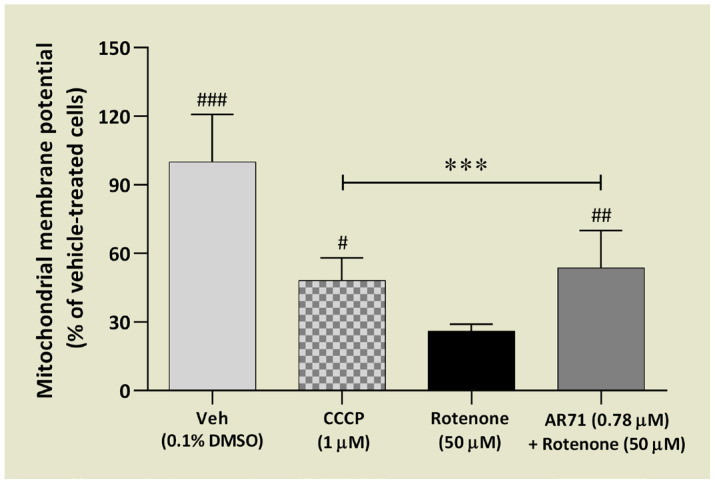
The effect of AR71 on mitochondrial membrane potential in rotenone-treated SH-SY5Y cells after 3 h. SH-SY5Y cells were pre-treated with AR71 (0.78 μM) for 1 h before rotenone stimulation (50 μM) for another 3 h. The positive control was 1 μM of CCCP (carbonyl cyanide m-chlorophenylhydrazone), an uncoupling agent on the proton gradient. Each point represents the mean ± SEM of two independent experiments, each consisting of four replicates per treatment group. Data are presented as a percentage of control cells treated with vehicle (Veh, 0.1% DMSO). One-way ANOVA and post hoc Sidak’s multiple comparison test: *** *p* < 0.001 vs. Veh; ^#^ *p* < 0.05, ^##^ *p* < 0.01, ^###^ *p* < 0.001 vs. rotenone-treated cells).

Next, the effect of AR71 (0.78 μM) when co-treated with rotenone was tested; 1 μM carbonyl cyanide m-chlorophenylhydrazone (CCCP) was used as a positive control. Although AR71 minimised the loss of MMP by 20% compared with the rotenone-treated cells, it did not restore MMP to the control level, which was still lower by half compared with the control cells (Figure 12). 

The presented results well document the neuroprotective effect of AR71 on the neuroblastoma cell line treated with rotenone. These findings are particularly intriguing in the context of PD therapy. Therefore, it would be logical to explore the therapeutic potential of AR71 in an animal model of PD.

### 2.6. Assessment of the Anti-Inflammatory Activity of AR71

**Figure 13:**
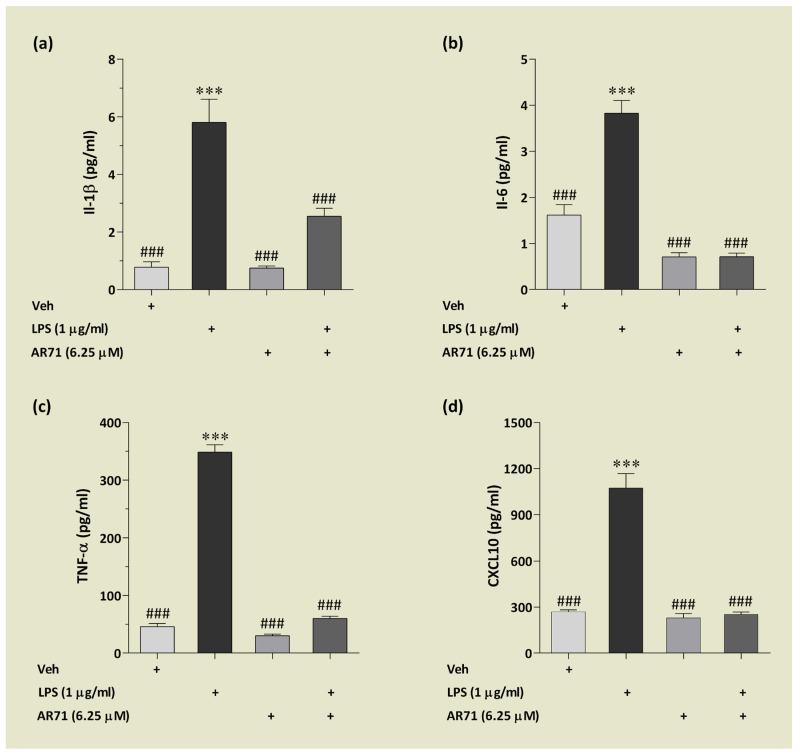
The effect of AR71 on the inflammatory response in BV-2 cells after LPS stimulation. Pro-inflammatory cytokines ((**a**) IL-1β, (**b**) IL-6, (**c**) TNF-α) and chemokine ((**d**) CXCL10) were measured in a culture medium using an inflammation panel MILLIPLEX^®^ kit (MCYTOMAG-70K). Data are presented as the mean ± SEM of two independent experiments, each consisting of three replicates per treatment group (*n* = 6). All samples were analysed in duplicate on a Luminex 200 multiplexing instrument. One-way ANOVA with post hoc Sidak’s multiple comparison test: *** *p* < 0.001 vs. Veh; ^###^ *p* < 0.001 vs. LPS-treated cells.

**Figure 14:**
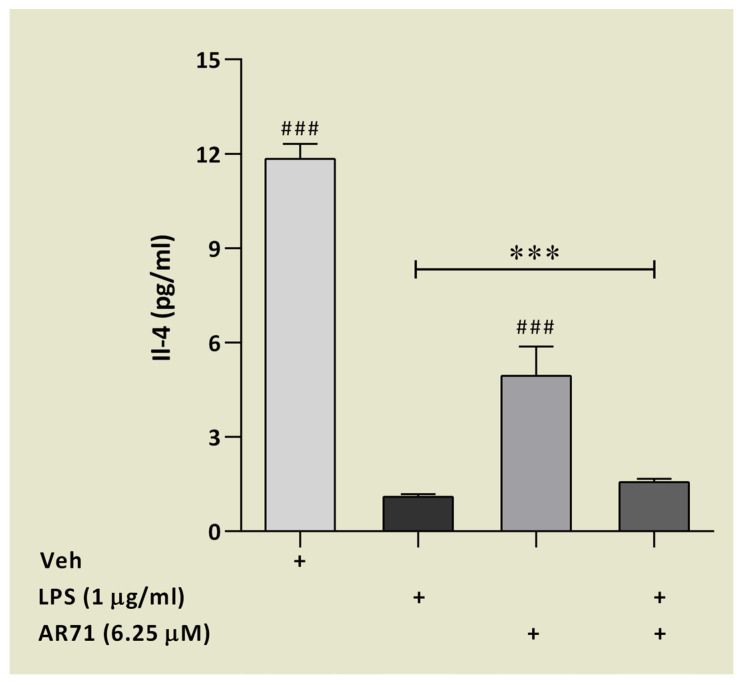
The effect of AR71 on the inflammatory response in BV-2 cells after LPS stimulation. An anti-inflammatory cytokine, IL-4, was measured in a culture medium using an inflammation panel MILLIPLEX^®^ kit (MCYTOMAG-70K). Data are presented as the mean ± SEM of two independent experiments, each consisting of three replicates per treatment group (*n* = 6). All samples were analysed in duplicate on a Luminex 200 multiplexing instrument. One-way ANOVA with post hoc Sidak’s multiple comparison test: *** *p* < 0.001 vs. Veh; ^###^ *p* < 0.001 vs. LPS-treated cells.

We previously reported that AR71 reduced the level of proinflammatory cytokines (IL-1β, IL-6, TNF-α) at both the level of mRNA (Real-Time PCR) and the protein level (ELISA) in an in vitro neuroinflammation model of LPS-treated BV-2 cells [41]. In the research presented in this paper, we extended the study of the anti-inflammatory properties of AR71 by analysing the panel of cytokines/chemokines in mouse microglia with the Luminex^®^ platform. We applied MILLIPLEX^®^ Magnetic Bead Panel that consists of nine different analytes regarding inflammation. The results are presented in Figure 13 and Figure 14. Some of the analytes were below the limit of quantification, and the reason for this is explained in the text. 

We found that BV-2 cells were unable to produce IFN-γ after stimulation with LPS; IFN-γ was below the limit of quantification (BLOQ). Similar results were also obtained by Kavanokuchi et al. [60], who demonstrated that the level of IFN-γ in supernatants from stimulated microglia was below the detection limit when assessed by an ELISA kit. It is acknowledged that activated T cells and natural killer cells primarily secrete IFN-γ [61]. In turn, the capability to produce IFN-γ was reported for microglia purified from the brains of adult SCID mice infected with Toxoplasma gondii that developed toxoplasmic encephalitis [62]. 

Regarding the capacity of AR71 to reduce inflammation in BV-2 cells, Figure 13 shows a clear trend in decreasing the level of three main pro-inflammatory cytokines. Compared with the cytokine level after LPS stimulation, the following reductions were observed: IL-1β was 2.66 times lower (Figure 13a), IL-6 was 5.94 times lower (Figure 13b), and TNF-α was 1.77 times lower (Figure 13c). Interestingly, these results were obtained with the Luminex^®^ platform but still correlate with those previously presented with the ELISA assay [41]. Additionally, we observed that another signalling molecule CXCL-10 (IP-10)—involved in initiating microglial activation—is repressed by AR71 in the presence of LPS (Figure 13d). It has been established that cytokines/chemokines released from chronically activated microglia could result in neuroinflammation, which in turn leads to neurodegeneration [63,64]. The results obtained in our study showing the downregulation of CXCL-10 in an in vitro model of LPS-induced neuroinflammation are extremely important since CXCL-10 is upregulated in AD and is associated with cognitive impairment in PD [65,66]. These experiments demonstrated that AR71 can alter the microglia phenotype. This fact encouraged us to conduct further studies to test markers specific to the neuroprotective phenotype of microglia. The results of these studies are presented in Figure 14. The level of IL-4 in unstimulated microglia ranged from almost 12 pg/mL, while after stimulation with LPS, the level dropped dramatically to 1 pg/mL. The treatment with AR71 alone resulted in half the IL-4 content compared with the unstimulated BV-2 cells. Unfortunately, the treatment with AR71 in the presence of LPS did not restore the level of IL-4 to the control point. 

Neuroinflammation is considered one of the common mechanisms in the pathogenesis of NDs [1,2,3,4,5,6]. The research conducted documented the anti-inflammatory activity of AR71. It is known that the compound combines the structure of an H_3_R blocker and a chalcone. There is abundant evidence linking histamine with the etiopathogenesis of neuroinflammation. Initially, mast cells’ participation in inflammatory CNS diseases was emphasised. However, more recent studies have provided evidence for the role of the central histamine system and, in particular, H_3_Rs. For example, progression of neuroinflammation was observed in H_3_-receptor knock-out mice [67]. H_3_Rs are thought to control brain histaminergic tone and act as gatekeepers for immune cell migration into the CNS. Studies also indicate that histamine may have a neuroprotective effect by interacting with H_1_R, H_2_R, and H_3_R present in astrocytes. This interaction leads to a decrease in the production of TNF-α and IL-1β, as well as an increase in the release of Glial Cell-Derived Neurotrophic Factor (GDNF) from these cells [68]. For this reason, the brain histamine system, mainly H_3_R, is being considered a target for developing new therapeutic strategies to treat neuroinflammatory and neurodegenerative diseases [69].

### 2.7. Effects of AR71 on Neuropathic Pain Symptoms in CCI-Exposed Mice

**Figure 15:**
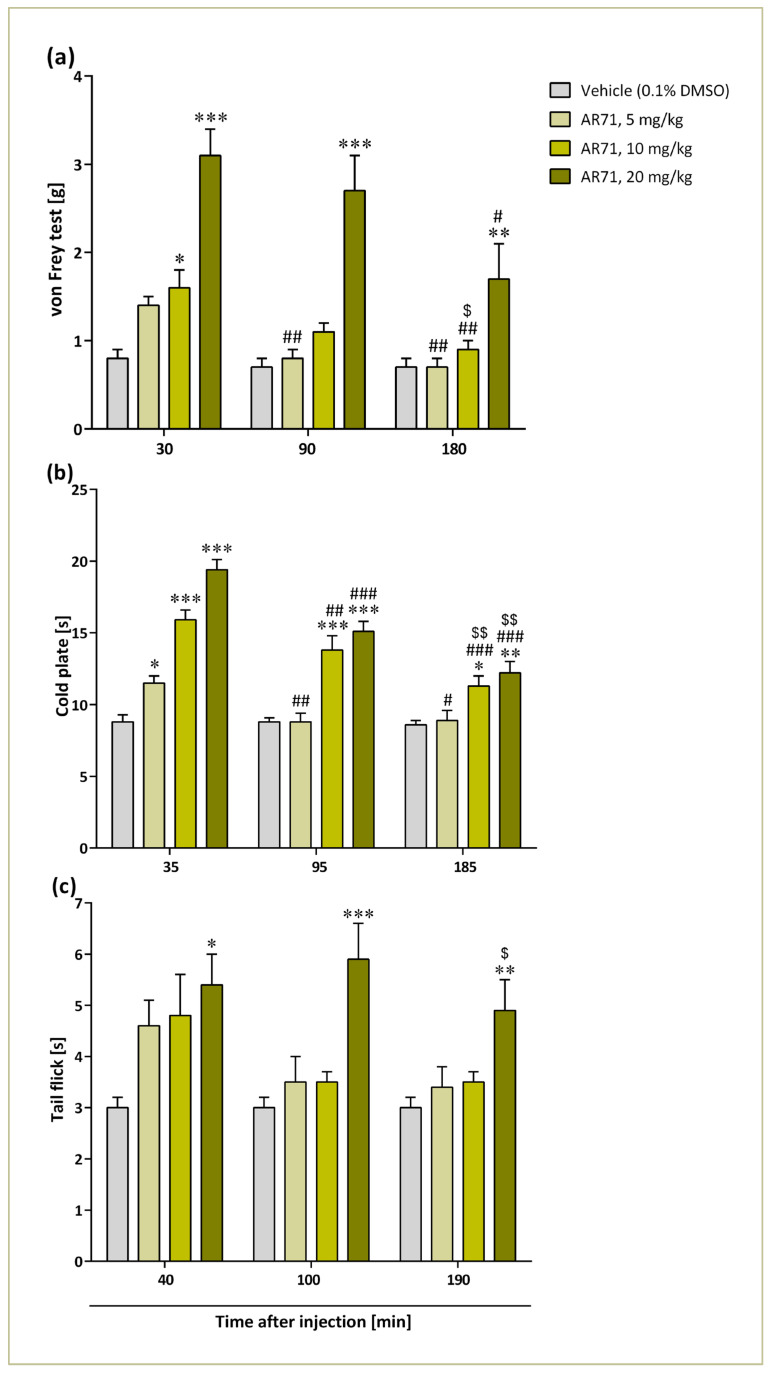
The effects of single i.p. administration of AR-71 (5, 10, and 20 mg/kg) on mechanical ((**a**), von Frey test) and thermal; (**b**), cold plate test; (**c**), tail flick test) stimulus on day 14 following CCI to the sciatic nerve were evaluated (*n* = 7–8 animals per group). Intergroup differences at each time point were analysed by one-way ANOVA with Bonferroni’s multiple comparison post hoc test. * *p* < 0.05, ** *p* < 0.01, *** *p* < 0.001 vs. the vehicle-treated group. Differences within the dose over time were analysed by one-way ANOVA with repeated measurements and then with Bonferroni’s multiple comparison post hoc test: ^#^ *p* < 0.05, ^##^ *p* < 0.01, ^###^ *p* < 0.001—2nd or 3rd time point vs. 1st time point; ^$^ *p* < 0.05, ^$$^ *p* < 0.01—3rd time point vs. 2nd time point.

The effects of a single intraperitoneal (i.p.) injection of AR71 at doses of 5, 10, and 20 mg/kg on mechanical (von Frey test) and thermal (cold plate and tail flick tests) stimulus were evaluated on day 14 following sciatic nerve injury (Figure 15). There were seven to eight animals per group. All these tests were performed in three time series after the administration of the compound. In the case of the von Frey test—after 30, 90, and 180 min; the cold plate test—after 35, 95, and 185 min; and the tail flick test—after 40, 100, and 190 min.

The mice were randomly divided into four experimental groups. One group received a vehicle treatment (10% DMSO/10%(2-Hydroxypropyl)-β-cyclodextrin/water for injection, i.p.), while the other three groups were administered with AR71 at doses of 5, 10, or 20 mg/kg of body weight (b.w.), i.p. Baseline measurements for all tests were conducted before the compounds were injected, and the results are presented as percentages of the maximal possible effect (% MPE). During all experiments, no sedative, locomotor, or gastrointestinal effects of AR71 were observed at any of the doses tested. All data from behavioural tests were analysed using a one-way ANOVA with Bonferroni’s multiple comparison post hoc test. 

In the von Frey test, we observed a significant impact of the tested compound at all time points (Figure 15a). In mice exposed to CCI, AR71 showed significant pain-relieving effects 30 min after injection of 10 mg/kg (*p* < 0.05) and 20 mg/kg (*p* < 0.001) in the von Frey test compared with the animals that received only vehicle. However, at time points 90 and 180 min after a single compound injection, only the highest dose (i.e., 20 mg/kg b.w.) increased withdrawal thresholds (*p* < 0.001 and *p* < 0.01, respectively) in the von Frey test. In this study, a one-way ANOVA with Bonferroni’s multiple comparison post hoc test revealed significant differences 90 min after injecting AR71 at a dose of 5 mg/kg b.w., as well as at all doses 180 min after administration, compared with the initial time point (30 min). Additionally, the 10 mg/kg b.w. dose was more effective 180 min after administration than 90 min (*p* < 0.05). 

Based on the data presented in Figure 15b, we observed a significant impact of AR71 treatment on thermal stimuli as measured in a cold plate test at all time points tested. Analgesic effects of a single AR71 injection were observed at 35 min for all doses tested compared with the control animals (5 mg/kg: *p* < 0.05; 10 and 20 mg/kg: *p* < 0.001). However, 95 and 185 min after injection, only the two higher doses were effective (95 min: 10 mg/kg and 20 mg/kg—*p* < 0.001; 185 min: 10 mg/kg—*p* < 0.05, 20 mg/kg—*p* < 0.01). At the 95 and 185 min time points, statistically significant differences were observed for all doses of AR71 compared with the results obtained 35 min after drug application. Furthermore, at the third time point (185 min), the analysis showed a better analgesic effect for 10 and 20 mg/kg b.w. doses compared with the second time (95 min). 

The results of the tail flick test indicated a significant analgesic effect of AR71 at the 20 mg/kg b.w. dose (*p* < 0.05) 40 min post-injection compared with the vehicle-treated mice (Figure 15c). At later time points (100 and 190 min), only the highest dose of the compound (20 mg/kg b.w.) showed significant pain-relieving effects (*p* < 0.001 and *p* < 0.01, respectively). Additionally, a statistically significant difference was observed at the 190 min mark after injection with AR71 for the same dose compared with the earlier time points (*p* < 0.05).

The mechanisms of neuropathic pain are complex and multifaceted. Several pathophysiological and biochemical changes lead to structural and functional adjustments in the nervous system. This includes increased excitatory neurotransmitters and neuropeptides such as histamine, bradykinin, serotonin, and glutamate [70]. The analgesic effect of AR71 was evaluated using a well-established experimental model of neuropathic pain (CCI) as well as tests assessing sensitivity to mechanical (the von Frey test) and thermal pain stimuli (the cold plate and tail flick tests) [29,71]. The CCI model reflects some of the clinical features of neuropathic pain, such as allodynia and hyperalgesia, in preclinical experimental settings [70]. 

The analgesic effects of AR71 were most effective at the highest dose (20 mg/kg b.w.) and within 30–45 min after injection. However, statistically significant differences from the control (Veh) were also observed at the second and third time points (Figure 15a–c). This may suggest that AR71 must be administered more than once, in repeated doses, to obtain a lasting analgesic effect. 

AR71’s analgesic effect may result from its ability to modulate histaminergic transmission (through interaction with the receptor) [28,29,70] and/or from the pharmacological activity of the chalcone moiety [34,35]. Our recent studies [29,72] revealed that the H_3_R receptor shows great promise as a target for developing new analgesic medications. Additionally, it can be used as a co-adjuvant in pain therapy [29]. It has previously been observed that H_3_R antagonists GSK189254 and GSK334429 are effective in rat models of neuropathic pain, whether surgically or virus-induced. Also, antagonism of supraspinal histamine H_3_R receptors modulates spinal neuronal activity in neuropathic rats. [73,74]. 

Excitatory histamine receptor signalling in nociceptive pathways is associated with increased pain symptoms, while inhibition of histamine receptor signalling mainly results in neuroprotective and antinociceptive effects [29]. Different histamine receptor subtypes are expressed in presynaptic and postsynaptic neuronal membranes [75]. H_3_Rs are mainly found in presynaptic neurons and serve as autoreceptors or heteroreceptors. They regulate neurotransmitter release from axon terminals, providing positive or negative feedback into the synaptic cleft [19,20,21]. Histamine H_1_Rs and H_4_Rs are located postsynaptically. Unlike the other three types of histamine receptors, H_4_Rs are not overexpressed in the CNS and are characteristic of immune system cells [20,21]. The resulting excitatory or inhibitory physiological effect of histamine receptors depends on the action of the neurotransmitter and the subsequent cascade. Expression of H_3_Rs and H_4_Rs on the opposite sides of the synaptic cleft may contribute to their effects on neuropathic pain [70]. However, as mentioned earlier, the exact location of H_4_R in neurons is still controversial.

Using quantitative single-cell Ca^2+^ imaging, researchers demonstrated that histamine triggers a Ca^2+^ increase in a specific subset of sensory neurons (3–10%) by activating H_1_Rs and H_4_Rs while inhibiting H_3_R. The decreased threshold in response to H_3_R antagonism is thought to be responsible for the pain-relieving effect of H_3_R antagonists. This activation triggers H_1_Rs and H_4_Rs on sensory neurons, which excites histamine-sensitive afferents. As a result, this process may modulate pain sensitivity [76]. 

Both natural and synthetic chalcones are known to exhibit various pharmacological activities, including anti-inflammatory and anti-nociceptive effects, which have been extensively documented in both in vitro and in vivo studies [38,41,77]. According to the published data, the anti-inflammatory effect of chalcones is primarily caused by their ability to inhibit the activity and expression of the key inflammatory mediators including cyclooxygenase, prostaglandin E2, inducible NO synthase, and nuclear factor κB [34]. Analgesic effects following i.p. administration of chalcones in mice and Wistar rats in writhing and formalin-induced paw oedema tests were similar to the effects of celecoxib, the reference drug [77]; celecoxib is a type of non-steroidal anti-inflammatory drug (NSAID) that inhibits prostaglandin G/H synthase (cyclooxygenase; COX) and is commonly used to reduce pain and inflammation. Our initial in vitro studies showed that AR71 suppresses inflammatory processes in murine microglia cells (BV-2 cell line) by reducing pro-inflammatory cytokines (IL-1β, IL-6, TNF-α) at both transcription and translation levels [41]. 

Injuries or disorders of the somatosensory nervous system can lead to neuropathic pain, which is known to be more challenging to treat than other forms of chronic pain. Conventional drug-based treatments are often not effective in treating this type of pain, leading to higher healthcare costs and increased reliance on medications. The current lack of effective responses to various drug therapies for neuropathic pain poses a significant challenge, prompting the search for new targets to develop safe and effective treatments for this condition. 

Based on previous and current studies, AR71’s analgesic effect is likely due to the compound’s multidirectional activity, including its anti-inflammatory effect. Previous studies have shown the effectiveness of compounds acting via more than one pharmacological target, i.e., dual piperidine-based H_3_R and Sigma-1 receptor ligands, in reducing neuropathic pain [72]. AR71, which combines the desired properties, also appears to be promising in this regard.

## 3. Materials and Methods

### 3.1. Key Reagents

MTT (3-(4,5-dimethylthiazol-2-yl)-2,5-diphenyltetrazolium bromide, phosphate-buffered saline (PBS), resazurin sodium salt, and dimethyl sulfoxide (DMSO) were from Sigma-Aldrich (St. Louis, MO, USA). MTS (3-(4,5-dimethylthiazol-2-yl)-5-(3-carboxymethoxyphenyl)-2-(4-sulfophenyl)-2H-tetrazolium, CellTiter96^®^ AQueous One Solution Cell Proliferation Assay kit) was purchased from Promega (no. G3581, Promega, Madison, WI, USA). Rotenone was purchased from Cayman Chemical (no. 13995, Ann Arbor, MI, USA). The LDH assay (CytoTox-ONE™ Homogeneous Membrane Integrity Assay) was from Promega (no. G7891, Promega, Madison, WI, USA). 

### 3.2. Cyclic Adenosine Monophosphate (cAMP) Accumulation Assay

The intrinsic activity of AR71 was evaluated in a 3′,5′-cyclic adenosine monophosphate (cAMP) accumulation assay in human embryonic kidney (HEK) 293 cells, stably expressing the hH_3_R [42], using the LANCE Ultra cAMP kit (Perkin Elmer, Waltham, MA, USA). The applied method was intended to measure the levels of cAMP generated because of changes in adenylyl cyclase activity mediated by GPCRs. The assay is based on the competition between the europium (Eu) chelate-labelled cAMP tracer and sample cAMP for binding sites on cAMP-specific monoclonal antibodies labelled with the ULight dye. The effect of AR71 (concentrations: 10^−10^–10^−5^ M) on reversing the inhibition of cAMP production by (R)-(-)-α-methyl histamine (30 nM) in forskolin-stimulated (10 μM) cells was evaluated. The experiment was conducted according to the principles described earlier [72]. Experiments were performed three times in triplicates, and IC_50_ values were determined using GraphPad Prism 8.4.3 software.

### 3.3. In Silico Studies 

Molecular docking was carried out using Schrodinger 2022-4 [78]. Bioactive conformers were generated using ConfGen [79] (water environment at physiological pH; only the 5 lowest energy conformers were chosen). Docking was performed according to standard protocol [80,81] (cubical box of Å, extra precision) and was validated beforehand by redocking native ligands to the corresponding receptors’ structures. Induced-fit docking was conducted using the Glide IFD module [82]. The putative binding energy of ligands (dG) was calculated using Prime MM-GBSA [83].

Dynamics simulations for generated complexes (T = 300 K, 1000 frames) were run in Desmond [84] for 100 ns. Additionally, 250 ns simulations were performed for the AR71-7F61 complex (H_3_R). The protein orientation in the membrane was obtained from the OPM database [85]. The TIP3P solvent model [86] and POPC membrane were applied. Selected frames were analysed using the Simulation Interaction Diagram tool and visual assessment. The figures shown are from the Maestro Schrödinger package.

### 3.4. In Vitro Evaluation of AR71 Metabolic Stability

Metabolic stability was assessed in human liver microsomes (HLMs, Sigma-Aldrich, St. Louis, MO, USA), as previously described [87,88]. After 120 min of incubation with HLMs, liquid chromatography–mass spectrometry (Waters ACQUITY TQD system with the TQ Detector, Waters, Milford, MA, USA) was used to identify the formed metabolites. MetaSite 6.0.1 software (Molecular Discovery Ltd., Hertfordshire, U.K.) was used to predict the most probable sites of metabolism in silico.

### 3.5. Cell Cultures 

HEK 293 cells, a human embryonic kidney cell line, were provided by Dr Thierry Calmels from Bioprojet-Biotech (France).

The astrocyte cell line from the human cerebral cortex was purchased from ScienCell Research Laboratories (San Diego, CA, USA; Cat no. 1800) and maintained according to the protocol recommended by the company (the ScienCell Research Laboratories’ protocol).

Human PBMCs (peripheral blood mononuclear cells) were isolated from a leucocyte-buffy coat collected from the blood of healthy non-smoking donors from the Blood Bank in Lodz, Poland.

SH-SY5Y cells (the human neuroblastoma cell line) were purchased from the American Type Culture Collection (ATCC^®^ no. CRL-2266™; Manassas, VA, USA). The cells were grown in a complete culture medium containing DMEM/F-12 medium (Gibco no. A41920-01) supplemented with 10% foetal bovine serum (FBS, Gibco no. 10500-064), non-essential amino acids (Biowest, Nuaillé, France), and antibiotics (Life Technologies) in a humidified incubator containing 95% air and 5% CO_2_ at 37 °C. Experiments were performed with SH-SY5Y cells from passage (15 up to 25—W.W.).

BV-2 cells (the murine microglial cell line) were purchased from the Interlab Cell Line Collection (ICLC) cell bank (ICLC, Genova, Italy). The cells were cultured in DMEM medium (Gibco no. 61965-026, ThermoFisher Scientific, Waltham, MA, USA) containing 10% charcoal-stripped foetal bovine serum (CS-FBS, Biowest no. S181f, batch no. S00NP) in a humidified incubator containing 95% air and 5% CO_2_ at 37 °C. CS-FBS was used to study the effect of compounds on neuroinflammation in vitro without the confounding effects of some cytokines that are naturally present in serum. Experiments were carried out with BV-2 cells from passages 7 to 13.

### 3.6. Cytotoxicity Assessment of AR71

#### 3.6.1. Effect of AR71 on the Viability of Human Astrocytes (MTT Assay)

The colorimetric MTT assay using 3-(4,5-dimethylthiazol-2-yl)-2,5-diphenyltetrazolium bromide (MTT; Sigma-Aldrich Chemical Co. Ltd., Saint Louis, MO, USA) was used for evaluation of astrocytes viability. The experiment was carried out following the procedure described in detail previously [17,89]. Astrocytes were seeded onto 96-well plates at a final density of 10 × 10^3^ cells/well. Following 24 h of culture, the cells were treated with AR71 (0.78–100 μM) for 4, 24, 48, or 72 h. After the respective incubation periods, MTT solution was added to the cell culture for another four hours. Absorbance was measured at 570 nm using a BioTek EL 800× microplate reader (BioTek, Winooski, VT, USA), with the value directly proportional to the number of viable cells. Cell viability was calculated by the following formula: Viability [%] = (A/AC) × 100%, where A represents the absorbance of the investigated sample and AC represents the absorbance of the negative control (untreated cells). The positive control (vehicle) was 0.1% DMSO.

#### 3.6.2. Effect of AR71 on Human Peripheral Blood Mononuclear Cell (PBMC) Viability

PBMCs were isolated from a leucocyte-buffy coat collected from the blood of healthy non-smoking donors from the Blood Bank in Lodz, Poland. They included lymphocytes, monocytes, and other white blood cells with a round nucleus. A portion of the leucocyte-buffy coat was diluted in 1% phosphate buffer saline (PBS). Then, it was centrifuged in a density gradient of Lymphosep (Cytogen, Zgierz, Poland) at 200× *g* for 20 min at room temperature. Next, the cells were collected and washed thrice in 1% PBS. The supernatant was poured off, and the pellet of the cells was resuspended in RPMI 1640 medium (Lonza, Basel, Switzerland) [90]. The study protocol was approved by the Committee for Research on Human Subjects of the University of Lodz, Poland (17/KBBN-UŁ/III/2019). 

The resazurin reduction assay was performed similarly to that previously described by O’Brien et al. [91]. Resazurin salt powder was dissolved in sterile PBS. The cells were seeded on 96-well plates in 5 × 10^4^ for PBMCs per well. AR71 was added to the wells to achieve final concentrations of 0.78, 1.56, 3.125, 6.25, 12.5, 25, 50, and 100 µM. In the next step, the plates were incubated at 37 °C in 5% CO_2_ for 2 h, 24 h, 48 h, and 72 h. Then, 10 µL of resazurin salt was added to each well, and the plates were incubated again at 37 °C in 5% CO_2_ for 2 h. Next, fluorescence was measured with microplate reader BioTek Synergy HT (Agilent Technologies, Inc., Santa Clara, CA, USA) using an excitation wavelength of 530/25 nm and an emission wavelength of 590/35 nm. 

The viability of PBMCs was estimated after 2, 24, 48, and 72 h of incubation with AR71. The viability of individual samples was calculated relative to the negative control (NC, untreated cells), which was taken as 100%. The positive control was PBMCs incubated with 0.1% DMSO (vehicle, Veh). The results represented the means with SEMs of six independent experiments and were expressed as a percentage of untreated control cells. The values were plotted against all used concentrations of AR71 to calculate the viability inhibition concentration at 50% (IC_50_) using GraphPad Prism 6.07 (GraphPad Software, Inc., San Diego, CA, USA).

#### 3.6.3. Effect of AR71 on the Viability of the Human Neuroblastoma (SH-SY5Y) Cell Line 

Neurotoxicity evaluation was performed with the SH-SY5Y cell line. The cells were seeded (5 × 10^3^ cells/100 μL/well) in transparent 96-well plates (Corning Falcon, no. 353072, Merck KGaA, Darmstadt, Germany), in a complete culture medium and cultured overnight. The next day, the medium was removed and replaced with a fresh one containing 0.1% DMSO (vehicle control, Veh) or increasing concentration of the AR71 compound (the concentrations studied covered the range of nanomolar and micromolar concentrations). Treatment with the compounds was performed for 24 h. At the end of the incubation time, the cells were examined under an inverted microscope to rule out possible precipitation of the compound in the culture medium. Cell viability was examined using an MTS-based CellTiter96^®^ AQueous One Solution Cell Proliferation Assay (Promega, Madison, WI, USA) following the manufacturer’s protocol. Briefly, 20 μL of MTS solution was pipetted directly into each well containing 100 μL of culture medium with or without cells (blank) and incubated at 37 °C for 1 h. The absorbance was measured at 490 nm using the Tecan Spark multimode plate reader (Tecan, Männedorf, Switzerland). A reference wavelength of 630 nm was used to subtract the background. The viability of individual samples was calculated relative to the vehicle-treated cells (0.1% DMSO). The results are presented as means with SEMs of two independent experiments, each comprising four replicates per treatment group. Data from the MTS assay were used to determine the IC_50_ value. It was calculated by fitting a nonlinear regression to a sigmoidal dose–response curve in GraphPad Prism version 8.0.1 (GraphPad Software, Inc., San Diego, CA, USA).

#### 3.6.4. Assessment of the Effect of AR71 on the Growth Rate of SH-SY5Y Cells (High Content Analysis Image Cytometry)

The principles of experimental design for evaluating the cytotoxicity of the examined compound using image cytometry have been previously described [92,93]. 

SH-SY5Y cells were initially seeded in 96-well plates at a density of 5000 cells per well in 120 μL of medium. After 24 h, the cells were exposed to different concentrations of AR71 for either 24 or 72 h. Following a wash with PBS, the cells were fixed with 4% formaldehyde for 20 min and then stained with 1 µg/mL of Hoechst 33342 for 30 min at room temperature. Subsequently, images of 16 fields per well were captured using the ArrayScan VTI HCS Reader from Thermo Fisher Scientific, Inc. (Thermo Fisher Scientific, Waltham, MA, USA), which was equipped with a 5× objective. The cell number was calculated as the sum of the Hoechst-33342-stained cell nuclei using Cell Cycle Bioapplication V3 software and expressed as a per cent of control. The remaining adherent cells in culture wells reflected the cytotoxic potential of the tested compound. In the next step, a series of detailed images were acquired using the ArrayScan VTI HCS Reader equipped with a 10× objective to collect at least 500 nuclei/cells per replicate. The DNA cell content of single cells was analysed based on Hoechst-33342 fluorescence intensity of their nuclei using Cell Cycle Bioapplication V3 software and presented as the DNA 2N/4N ratio. All experiments were performed three times, each in four replicates. IC_50_ values were calculated with a non-linear fit to a sigmoidal dose–response curve (log compound vs. normalised response) using GraphPad Prism 6.07. The results are presented as mean ± SEM from at least three independent experiments. Statistical significance was evaluated using one-way ANOVA followed by Dunnett’s test. * *p* < 0.05, ** *p* < 0.01, and *** *p* < 0.001 indicate significant differences compared with the control counterparts. 

### 3.7. Assessment of Neuroprotective Properties of AR71

#### 3.7.1. Lactate Dehydrogenase Leakage Assay (LDH Assay)

The protective effect of AR71 against rotenone-induced toxicity in SH-SY5Y cells was assessed by measuring LDH release. SH-SY5Y cells (6 × 10^3^ cells/100 μL/well) were seeded in black 96-well plates Nunclon^TM^ Delta Surface no. 137101 (ThermoFischer Scientific, Waltham, MA, USA) and cultured overnight. The next day, the medium was removed and replaced with a fresh one with an increasing concentration of AR71 (0.0975–50 μM). After 1 h of compound pre-treatment, the neurotoxin rotenone (6.25 μM) was added, and the cells were cultured for another 24 h. For the negative and positive controls, cells were treated with 0.1% DMSO and 6.25 μM of rotenone alone, respectively. The cells treated with the compound alone were used to compare the effect with those co-treated with rotenone. After 24 h, the level of LDH leakage was measured by the CytoTox-ONE™ Homogeneous Membrane Integrity Assay (Promega, Madison, WI, USA) following the manufacturer’s protocol. Briefly, 100 μL of CytoTox-ONE™ reagent (Promega, Madison, WI, USA) was added to each well, and the cells were incubated for 10 min at room temperature protected from light. The fluorescence was measured using the multimode plate reader Tecan Spark (Tecan, Männedorf, Switzerland) with an excitation wavelength of 560 nm and an emission wavelength of 590 nm.

#### 3.7.2. Mitochondrial Membrane Potential (MMP) 

The MMP was measured by a commercially available kit (Sigma Aldrich, MAK159, for live cells) that utilises a cationic, lipophilic dye JC-10. It allows for ratiometric analysis of mitochondrial membrane potential, where a shift from orange (λEX/λEM: 540 nm/590 nm) to green fluorescence (λEX/λEM: 490 nm/525 nm) is indicative of compromised mitochondria. For this assay, the SH-SY5Y cells (1 × 10^4^ cells/well) were seeded in a black-sided clear bottom 96-well plate (Thermo Scientific Nunclon^TM^ Delta Surface no. 137101, Denmark) in a complete culture medium. First, to choose the optimal concentration of rotenone in this assay, a dose–response effect of rotenone (0.05, 0.5, 5, 50 μM) was studied. Next, the cells were pre-treated with AR71 (0.78 μM) for 1 h before rotenone stimulation (50 μM) for another 3 h. AR71 at a concentration of 0.78 μM did not affect mitochondrial membrane potential in SH-SY5Y cells. The positive control was carbonyl cyanide m-chlorophenylhydrazone (CCCP; 1 μM)—uncoupling agent on the proton gradient. After incubation, 50 μL/well of the JC-10 dye loading solution was added to each well with cells. The plate was incubated for 45 min in the incubator. Then, 50 μL/well of Assay Buffer B was added to each well, and the fluorescence was monitored immediately. The red/green fluorescence intensity ratio was used to calculate the results. 

### 3.8. Assessment of Anti-Inflammatory Activity of AR71 (Luminex^®^ Immunoassay)

The Luminex assay is a form of immunoassay that utilises magnetic microparticles and the same sandwiching techniques as ELISAs. To analyse the panel of cytokines/chemokines in mouse microglia, we used a MILLIPLEX^®^ Magnetic Bead Panel (MCYTOMAG-70K, Millipore, Sigma-Aldrich, Darmstadt, Germany). The following analytes were studied: IFN-γ, IL-1β, IL-4, IL-6, IL-10, IL-12 (p40), IP-10, MIP-2, and TNF-α. The preparation of antibody-immobilised beads, quality controls, mouse cytokine standard, and immunoassay procedure, were performed according to the manufacturer’s protocol. All standards, quality controls, and samples were analysed in duplicate on the Luminex 200 multiplexing instrument (Luminex Corp, Austin, TX, USA). Sample analyte concentrations were determined using Belysa 1.1.0 curve-fitting software and the standard curve data from each run (Millipore, Sigma-Aldrich, Darmstadt, Germany). To perform multiplex cytokine analysis, BV-2 cells (150 × 10^3^ cells/well/500 μL in 24-well plate) were pre-treated for 1 h with AR71, and then LPS (1 μg/mL) was added to induce inflammation. The cells were cultured for another 24 h. After this time, cell culture supernatants were collected, centrifuged to remove cell debris, and stored at −80 °C for analysis. To avoid multiple freeze/thaw cycles, the samples were aliquoted. 

### 3.9. Pharmacological Studies In Vivo—Assessment of the Analgesic Activity of AR71

Assessment of the analgesic activity of AR71 was performed using a mouse experimental model of chronic constriction injury of the sciatic nerve [71], by three tests including the von Frey test, the cold plate test, and the tail flick test. 

#### 3.9.1. Animals

All animal procedures were completed following the recommendations of the International Association for the Study of Pain [94] and the NIH Guide for the Care and Use of Laboratory Animals. Experiments were approved by the II Local Ethics Committee Branch of the National Ethics Committee for Experiments on Animals based at the Maj Institute of Pharmacology, Polish Academy of Sciences, Kraków, Poland (approval number: 24/2022). Care was taken to minimise animal suffering and the number of animals used (3R policy). Adult male albino Swiss CD-1 mice (initially weighing 18–20 g, aged 6 weeks) were purchased from Charles River Laboratories (Hamburg, Germany). The animals were housed in groups of seven individuals under controlled conditions (temp. 21 ± 2 °C; 12 h light/dark cycle, lights on at 6:00 a.m.) with ad libitum food and water.

#### 3.9.2. Chronic Constriction Injury of the Sciatic Nerve (CCI)

The model of chronic constriction injury (CCI) to the sciatic nerve was established according to Bennett and Xie [71]. The surgical method was performed under isoflurate anaesthesia (2% isoflurane in 100% oxygen with a flow of 1.5 L/min) [29]. Below the right hipbone, a small incision was performed, and three ligatures (4/0 silk) around the sciatic nerve were made (with 1 mm spacing) until a brief twitch in the respective hind limb was observed. The CCI procedure leads to hypersensitivity to mechanical and thermal stimuli. Behavioural tests were performed on day 14 after the injury. AR71 was dissolved in 10% DMSO/10% (2-Hydroxypropyl)-β-cyclodextrin/water for injection and was given i.p. at doses of 5, 10, and 20 mg/kg of body weight (injection volume 10 mL/kg of bw). The control group received the vehicle (10% DMSO/10% (2-Hydroxypropyl)-β-cyclodextrin/water for injection). 

#### 3.9.3. Behavioural Tests 

Behavioural experiments were performed between 8:00 a.m. and 2:00 p.m. The results in AR71-treated mice were compared to those in the vehicle-treated control. Experiments were performed 30, 90, and 180 min (von Frey test); 35, 95, and 185 min (cold plate test); and 40, 100, and 190 min (tail flick test) after AR71 or vehicle injections, following the rules described earlier [29,72].

The von Frey test allowed us to evaluate mechanical hypersensitivity. The animals were housed in cages with wire mesh floors. The ipsilateral and contralateral sides of mice with CCI of the sciatic nerve responded (paw withdrawal, shaking, or licking) to a mechanical stimulator (calibrated nylon monofilaments: 0.6–6 g, Stoelting) in serial increments. 

The cold plate test (performed by the Cold/Hot Plate Analgesia Meter, Columbus Instruments, N. Hague Ave, Columbus, OH, USA) was used to assess sensitivity to noxious thermal stimuli. The plate temperature was maintained at 2 °C, and the cut-off delay was 30 s. The mice were placed on a cold plate, and the time it took to lift the injured paw was recorded.

The responsiveness to thermally induced pain was determined by a tail flick analgesia meter (Tail-Flick Unit, Ugo Basile, Gemonio, Italy) as previously described [29]. Tail flick latency was measured on the dorsal part of the tail at two-thirds of its length by applying a focused beam of light (thermal stimulus). The time interval between the thermal stimulus’s onset and the tail’s withdrawal from the beam was recorded. The cut-off latency was 9 s to prevent tissue damage. 

### 3.10. Statistical Analysis 

The results are presented as means ± standard errors of the mean (SEM). All statistical analyses were conducted using GraphPad Prism 6.07/8.01/8.4.3 software (GraphPad Software, Inc., San Diego, CA, USA). For in vivo and biochemical studies, statistical significance was determined using one-way ANOVA followed by post hoc Dunnett’s, Bonferroni’s, or Sidak’s multiple comparisons test. The significance levels were denoted as follows: *p* < 0.05 (*), *p* < 0.01 (**), and *p* < 0.001 (***). 

## 4. Conclusions

In summary, the future of therapy for NDs lies in new strategies targeting multiple pharmacological pathways. Our studies indicate that the activity of AR71, the compound combining a piperidinylpropoxy motif typical for H_3_R antagonists/inverse agonists with a chalcone moiety, appears promising in this regard. It can be concluded that AR71 (1) demonstrates good metabolic stability, (2) exhibits anti-inflammatory, neuroprotective, and analgesic properties, and (3) also shows antiproliferative activity in higher concentrations in the micromolar range. Thus, the results of the current studies on the pharmacological profile of AR71 suggest further testing its therapeutic potential in in vitro and in vivo studies, e.g., in animal models of ND and/or CNS cancer treatment.

## Figures and Tables

**Table 1:** The most probable metabolic pathways of AR71 after a 120 min reaction with human liver microsomes.

Compound *m*/*z*	Metabolite *m*/*z*	Metabolic Pathway
AR71 440.31 g/mol 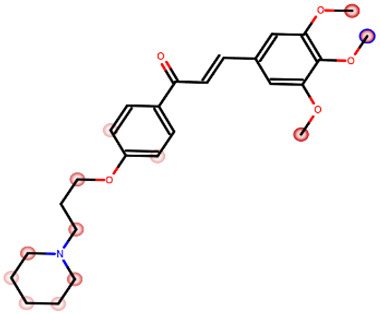	456.27 (M1)	hydroxylation
	355.30 (M2)	decomposition
	458.34 (M3)	hydroxylation/reduction
	438.32 (M4)	dehydrogenation
	426.28 (M5)	demethylation
	442.31 (M6)	demethylation/hydroxylation

Metabolic stability was conducted in human liver microsomes (HLMs). After incubating AR71 for 120 min with HLMs, liquid chromatography–mass spectrometry (LC-MS) was used to identify the formed metabolites. In the MetaSite prediction, the darker red colour in the structure means a higher probability of being involved in the metabolism pathway. The blue circle marks the site of the compound with the highest probability of metabolic bioconversion.

**Table 2:** IC_50_ values obtained for AR71 against human astrocytes and PBM cells.

Incubation Time	IC_50_ (μM)	
	Human Astrocytes	PBM Cells
2 h	41.28	70.99
24 h	20.21	20.98
48 h	12.53	7.48
72 h	9.69	4.53

IC_50_ values were determined after 2, 24, 48, or 72 h of incubating astrocytes or PBMCs with AR71 at doses of 0.78–100 μM (6 experiments).

## Data Availability

All data are contained within the article.

## Supplementary Materials

The following supporting information can be downloaded at https://www.mdpi.com/article/10.3390/ijms25158035/s1.
