# Supplementary material for: AR71, Histamine H3 Receptor Ligand—In Vitro and In Vivo Evaluation (Anti-Inflammatory Activity, Metabolic Stability, Toxicity, and Analgesic Action)
Source: Int J Mol Sci. 2024 Jul 23;25(15):8035. doi: 10.3390/ijms25158035 (PMC11311998; doi:10.3390/ijms25158035)
Supplement: Supplementary file 1 [file ijms-25-08035-s001.zip › Figure S1_AR71_cAMP.pdf]

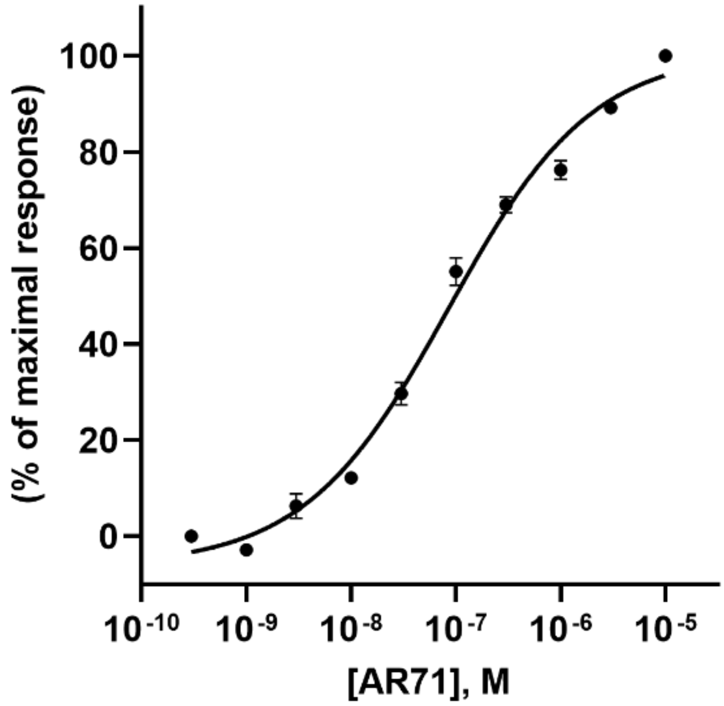

Figure S1. Forskolin-stimulated cAMP accumulation studies in HEK 293 cells expressing the human histamine H_3 receptor, co-treated with (R)(-)-α-Methylhistamine (10 μM) and AR71 (30 nM).
