# Supplementary material for: AR71, Histamine H3 Receptor Ligand—In Vitro and In Vivo Evaluation (Anti-Inflammatory Activity, Metabolic Stability, Toxicity, and Analgesic Action)
Source: Int J Mol Sci. 2024 Jul 23;25(15):8035. doi: 10.3390/ijms25158035 (PMC11311998; doi:10.3390/ijms25158035)
Supplement: Supplementary file 1 [file ijms-25-08035-s001.zip › Figure S3_H1R_Protein-Ligand contacts timeline.pdf]

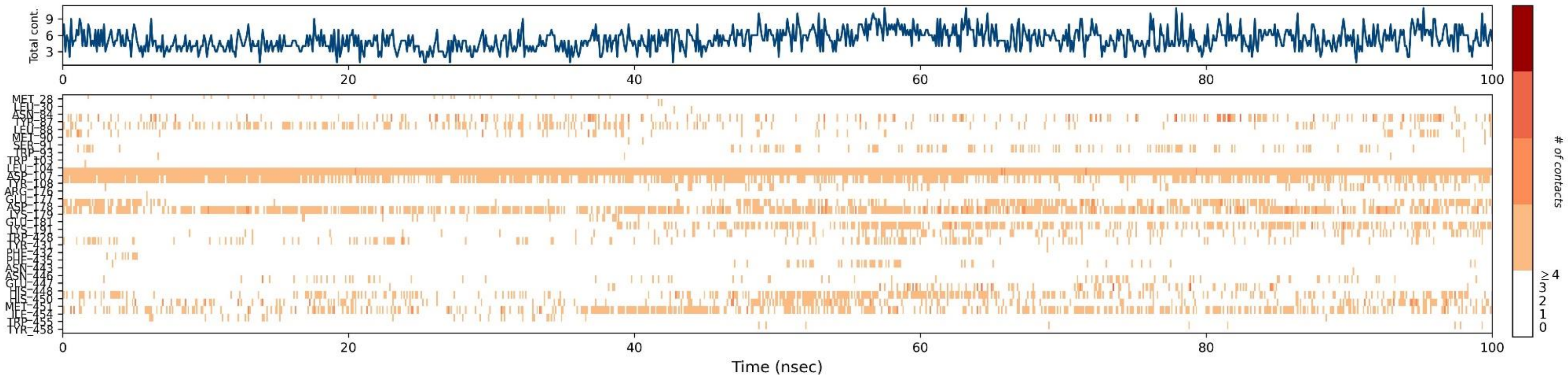

Figure S3. Protein-ligand contacts timeline for 100 ns simulation of AR71 in complex with histamine H1 receptor structure (3RZE); the darker colour, the more interactions recorded.
