# Supplementary material for: AR71, Histamine H3 Receptor Ligand—In Vitro and In Vivo Evaluation (Anti-Inflammatory Activity, Metabolic Stability, Toxicity, and Analgesic Action)
Source: Int J Mol Sci. 2024 Jul 23;25(15):8035. doi: 10.3390/ijms25158035 (PMC11311998; doi:10.3390/ijms25158035)
Supplement: Supplementary file 1 [file ijms-25-08035-s001.zip › Figure S4.a-d_Metabolic stability of AR71.pdf]

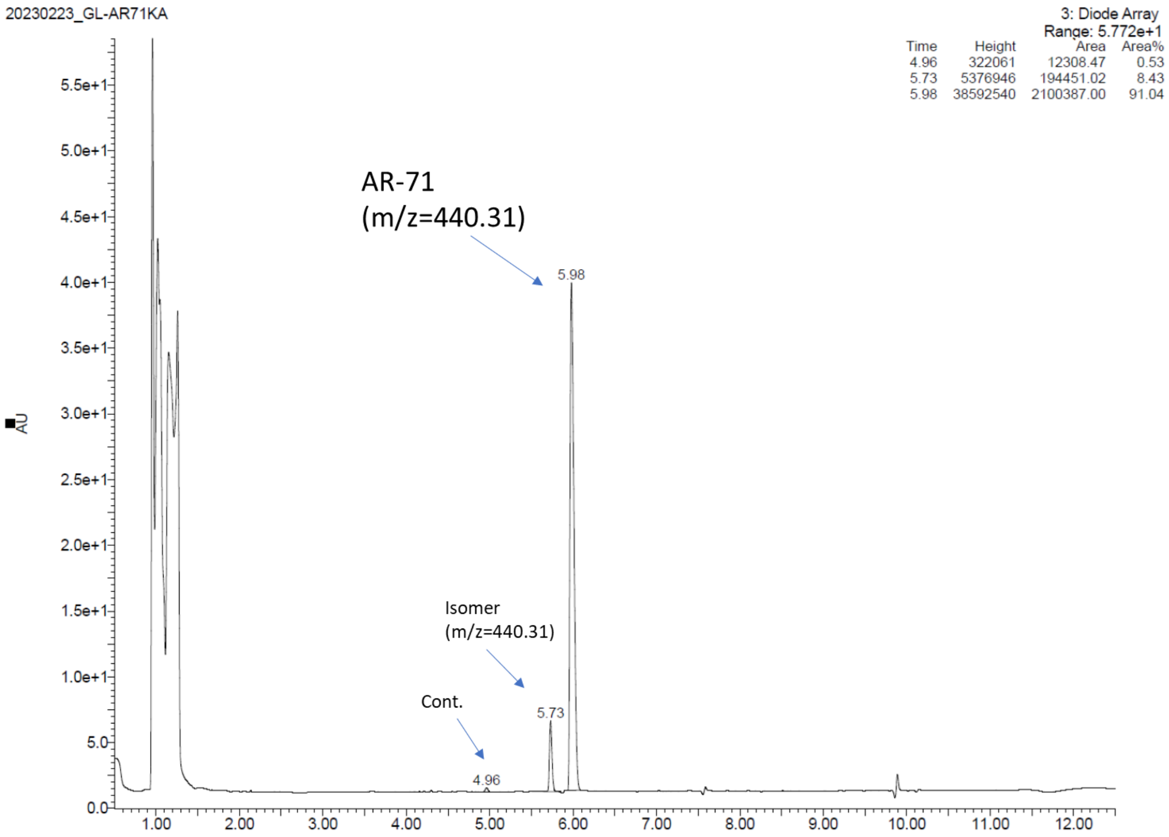

Figure S3.a. UPLC spectra after 120 min incubation of compound **AR-71** in TRIS buffer pH=7.4 at 37°C (**control reaction without microsomes**). The presence of isomer and small, unidentified contamination were found.

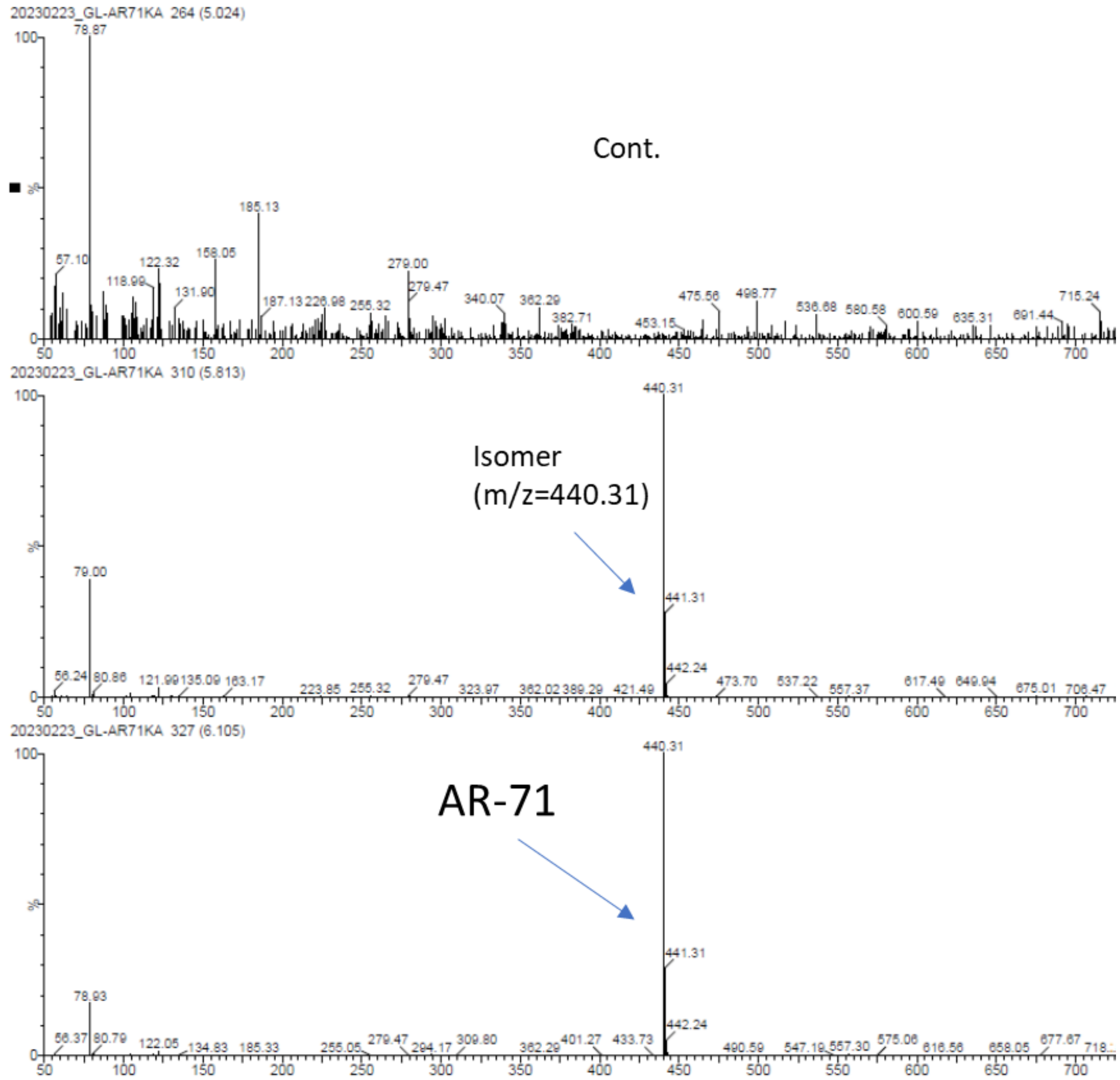

Figure S3.b. MS analyses of **AR-71,** isomer, and contamination from **control reaction**.

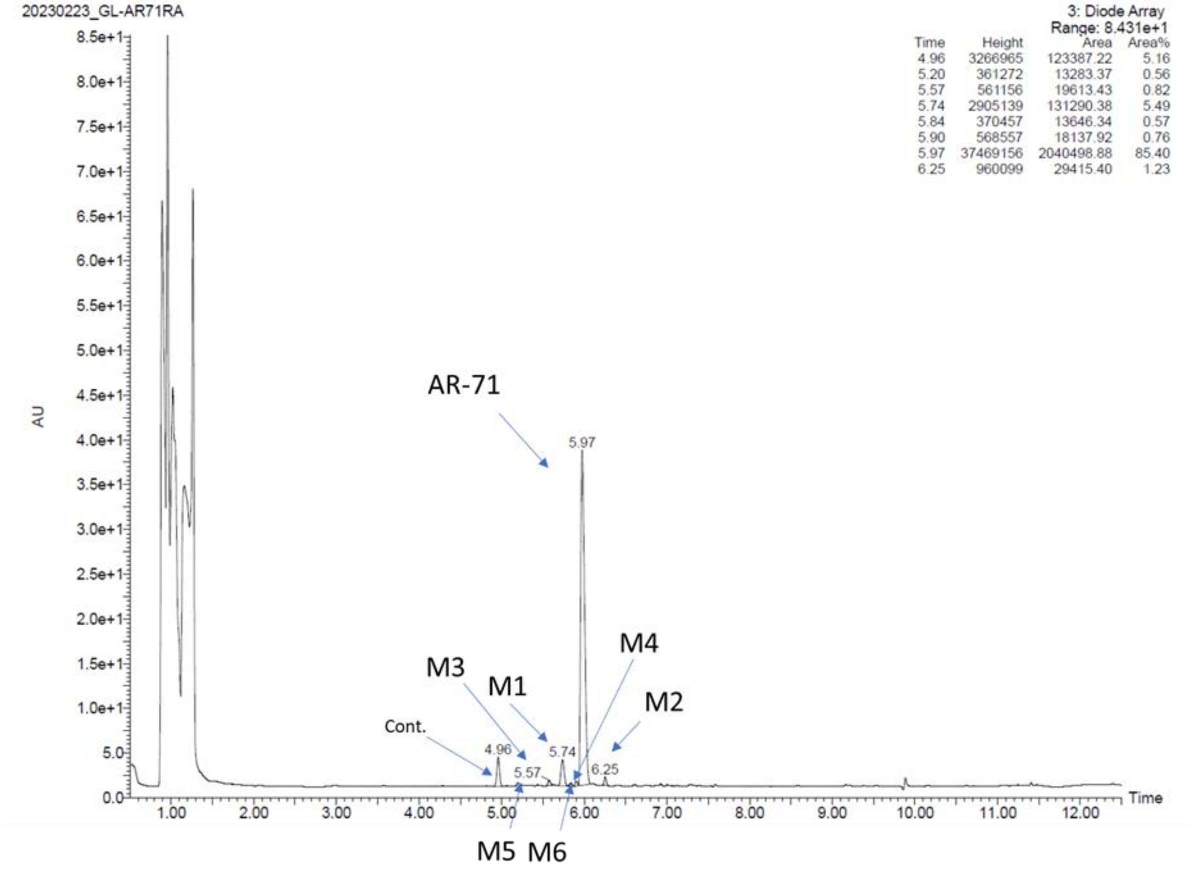

Figure S3.c. UPLC spectra after 120 min incubation of compound **AR-71 with human liver microsomes (HLMs)** in TRIS buffer pH=7.4 at 37ºC. Six metabolites were found (see Table 1). 85.40% of **AR-71** remained in the reaction mixture.

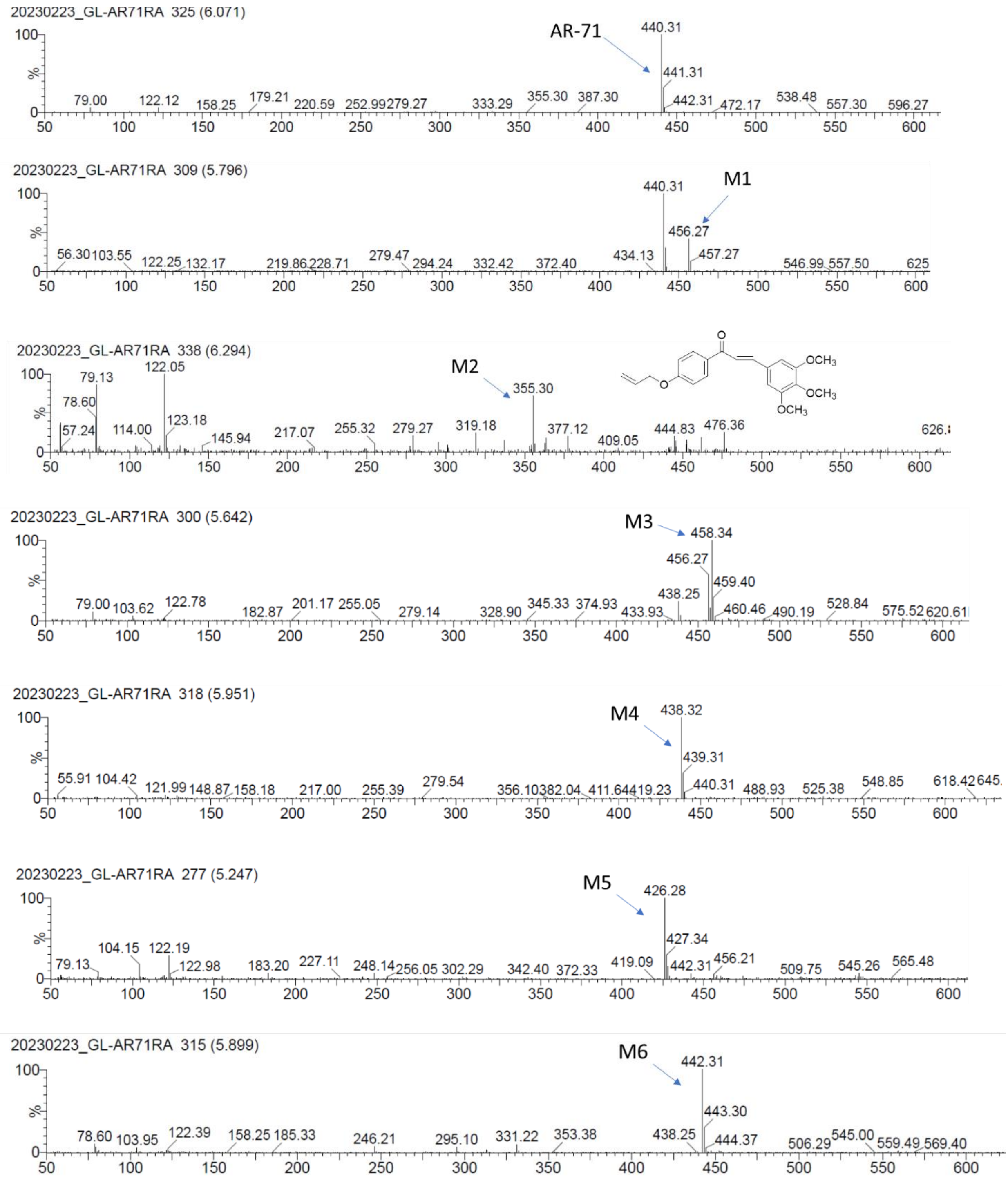

Figure S3.d. MS analyses of **AR-71** and its metabolites.
