# Supplementary material for: AR71, Histamine H3 Receptor Ligand—In Vitro and In Vivo Evaluation (Anti-Inflammatory Activity, Metabolic Stability, Toxicity, and Analgesic Action)
Source: Int J Mol Sci. 2024 Jul 23;25(15):8035. doi: 10.3390/ijms25158035 (PMC11311998; doi:10.3390/ijms25158035)
Supplement: Supplementary file 1 [file ijms-25-08035-s001.zip › Table S1_7F61_AR71_rmsd-per-residue.pdf]

Column1	Column2	Column3	Column4	Column5	Column6
Reference Residues	_:0(UNK)	A:27(ARG)	A:28(GLY)	A:29(PHE)	A:30(SER)
Structure2	4,11	4,73	6,79	8,13	6,09
Structure3	4,65	3,91	5,44	6,85	5,16
Structure4	6,07	7,97	7,89	7,08	5,75
Structure5	3,76	4,71	6,41	7,54	5,54
Structure6	4,69	3,99	3,99	4,37	5,39
Structure7	3,81	2,75	3,78	6,39	8,39
Structure8	3,04	2,7	4,3	7,39	7,75
Structure9	2,36	2,08	3,13	3,53	5,55
Structure10	2,14	2,48	2,16	2,03	5,05
Structure11	2,22	2,27	2,43	1,73	4,45
Structure12	4,36	4,88	5,89	7,72	5,96
Structure13	3,09	1,51	1,55	1,09	3,49
Structure14	3,53	2,12	1,78	0,91	1,77
Structure15	1,72	1,45	1,58	0,79	2,91
Structure16	1,63	1,25	1,07	0,94	2,09
Structure17	1,48	1,16	1,09	1,29	1,75
Structure18	3,14	1,72	1,24	1,19	2,27
Structure19	2,01	0,77	0,5	0,8	1,44
Structure20	1,33	2,18	1,44	1,16	2,05
Structure21	1,29	1,49	1,16	0,85	1,31
**mean RMSD**	**3,0215**	**2,806**	**3,181**	**3,589**	**4,208**

Column7	Column8	Column9	Column10	Column11	Column12
A:31(ALA)	A:32(ALA)	A:33(TRP)	A:34(THR)	A:35(ALA)	A:36(VAL)
4,47	4,69	4,97	3,85	3,84	4,33
3,56	4	4,8	3,6	3,53	3,84
6,68	6,96	4,71	2,41	1,64	2,71
4,07	4,43	4,95	3,67	3,85	4,34
3,89	4,41	3,64	2,88	2,58	2,82
5,45	5,93	5,98	3,23	3	3,52
6,25	6,34	5,93	2,23	1,5	2,98
4,56	5,32	5,61	2,22	1,84	1,98
1,8	1,4	3,18	0,73	0,65	1,51
2,06	2,05	4,27	0,61	0,55	2,43
4,34	5,44	5,9	4,1	4,37	4,8
1,02	0,82	2,51	1,63	2,09	2,09
2,02	3,72	6,02	1,52	1,41	2,33
1,59	2,22	3,1	1,89	2,05	1,92
1,38	2,32	3,84	1,77	2,16	2,23
1,11	2,31	3,49	1,91	2,31	2,44
1,3	2	2,78	1,82	2,56	2,61
0,85	1,99	3,28	2,06	2,79	2,99
1,17	2,38	5,07	1,28	0,81	1,97
1,3	1,17	1,29	1	1,14	0,84
**2,9435**	**3,495**	**4,266**	**2,2205**	**2,2335**	**2,734**

Column13	Column14	Column15	Column16	Column17	Column18
A:37(LEU)	A:38(ALA)	A:39(ALA)	A:40(LEU)	A:41(MET)	A:42(ALA)
3,46	3,13	3,06	2,77	2,11	2
2,84	2,32	2,1	2	1,16	0,88
3,15	2,86	2,24	2,29	2,53	2,49
3,37	3,02	2,82	2,51	2,05	1,76
2,64	2,94	2,51	2,19	2,29	2,08
3,12	2,8	2,58	2,11	2,16	2,15
2,06	1,23	1,19	1,67	1,36	1
1,69	1,41	1,18	1,39	0,92	0,98
1,28	0,83	0,65	1,84	1,06	1,14
1,83	1,17	1,07	1,59	1,49	1,19
4,09	3,91	3,6	3,55	2,84	3,1
1,69	1,53	1,43	1,31	1,56	1,85
2,65	1,63	0,93	1,14	0,94	1,09
1,39	0,83	0,94	1,35	1,28	1,6
1,4	1,06	0,93	1,71	1,21	1,71
1,86	1,56	1,29	0,96	0,63	0,53
1,66	1,29	1,31	1,68	1,81	1,92
1,92	1,39	1,38	1,45	0,88	0,8
2,27	0,89	0,77	1,23	1,24	0,64
1,2	1,1	1,38	1,46	0,92	1,02
**2,2785**	**1,845**	**1,668**	**1,81**	**1,522**	**1,4965**

Column19	Column20	Column21	Column22	Column23	Column24
A:43(LEU)	A:44(LEU)	A:45(ILE)	A:46(VAL)	A:47(ALA)	A:48(THR)
2,26	1,5	1,4	1,86	1,3	1,83
1,56	1,48	0,86	1,31	1,05	1,22
2,73	1,6	1,95	2,63	2,08	2
2,03	1,47	1,39	1,12	0,57	0,85
2,32	1,63	2,12	2,46	2,22	2,15
2,54	1,94	1,96	2,36	1,6	1,35
2,09	1,23	1,08	1,81	0,99	1,17
1,39	0,64	1,5	1,9	1,2	1,01
1,35	1,16	0,95	1,81	1,67	1,02
2,17	0,78	0,8	0,78	0,75	0,68
3,23	2,56	2,64	3,15	2,12	2,2
1,44	1,75	1,35	2,18	1,6	1,3
0,94	1,06	0,94	1,41	1,22	0,99
2,14	1,38	1,07	1,57	1,19	1,11
2,32	1,76	1,68	1,99	1,71	1,69
1,59	1,06	0,39	0,32	0,34	0,27
1,73	1,56	1,33	1,33	0,98	0,9
0,92	0,59	0,7	0,95	0,91	0,6
1,05	0,55	0,34	0,66	0,58	0,32
1,45	1,4	0,66	1,21	1,25	0,81
**1,8625**	**1,355**	**1,2555**	**1,6405**	**1,2665**	**1,1735**

Column25	Column26	Column27	Column28	Column29	Column30
A:49(VAL)	A:50(LEU)	A:51(GLY)	A:52(ASN)	A:53(ALA)	A:54(LEU)
1,76	2,27	1,63	1,31	1,39	1,07
1,54	1,54	1,25	1,32	1,23	0,9
1,61	3,17	1,98	1,23	1,78	1,81
1,48	1,48	1,33	1,24	1,69	1,26
2,32	2,2	2,03	1,77	1,87	2,14
1,71	1,95	1,41	1,43	1,96	2,07
1,49	2,52	1,05	1,1	1,07	1,75
1,02	1,25	0,91	0,87	1,43	1,6
1,28	1,16	0,93	1,37	1,56	1,59
1,04	1,14	0,89	1,08	1,2	1,65
2,05	2,9	2,21	1,88	2,75	2,03
1,18	1,46	0,93	1,34	1,59	2,05
1,32	1,13	0,36	1,04	1,06	1,07
0,86	0,65	0,66	0,81	0,83	1,11
1,83	1,9	1,48	1,58	1,54	1,8
0,37	0,74	0,46	0,48	0,53	0,61
0,97	0,59	0,46	0,97	0,93	1,13
0,88	1,8	1,06	0,83	1,29	1,63
0,42	0,47	0,67	0,92	0,75	0,88
0,75	0,81	0,53	0,85	1,14	1,37
**1,294**	**1,5565**	**1,1115**	**1,171**	**1,3795**	**1,476**

Column31	Column32	Column33	Column34	Column35	Column36
A:55(VAL)	A:56(MET)	A:57(LEU)	A:58(ALA)	A:59(PHE)	A:60(VAL)
0,85	1,38	2,13	2,15	2,22	3,07
0,68	1,61	2,05	1,15	1,68	2,82
1,61	2,06	2,21	1,6	2,39	3,54
0,84	1,53	1,32	1	1,56	1,48
1,63	1,72	2,19	1,99	1,4	1,74
1,6	1,73	2,44	2,22	1,66	2,59
0,81	1,01	1,32	1,14	1,13	2,45
1,3	1,4	2,06	2	1,67	1,78
1,41	1,7	1,78	1,19	1,4	1,97
1,35	1,23	1,66	1,45	1,3	1,44
1,72	2,67	2,9	2,77	2,7	3,77
2,29	2,02	2,16	2,06	2,19	2,07
1,61	1,4	2,11	1,57	1,67	1,85
1,68	1,67	1,54	1,19	0,74	1,01
1,18	1,37	1,75	1,56	1,51	1,96
0,65	0,67	0,75	0,39	0,95	0,83
0,89	1,14	1	0,83	1,12	0,62
1,11	1,11	1,87	1,32	1,61	1,87
0,71	0,61	1,11	0,78	0,51	0,99
0,72	0,61	1,19	0,81	0,88	1,19
**1,232**	**1,432**	**1,777**	**1,4585**	**1,5145**	**1,952**

Column37	Column38	Column39	Column40	Column41	Column42
A:61(ALA)	A:62(ASP)	A:63(SER)	A:64(SER)	A:65(LEU)	A:66(ARG)
2,59	2,68	2,57	3,02	2,24	3,03
1,5	1,56	1,24	1,78	1,52	2,07
3,43	3,17	3,03	3,67	1,85	3,74
1,44	1,36	1,27	2,24	1,34	1,96
2,03	1,97	1,64	2,65	1,81	1,8
2,48	2,3	1,91	2,42	1,85	4,12
1,39	1,51	1,2	1,54	1,42	3,38
1,83	1,92	1,98	2,04	2,41	1,71
1,3	1,66	1,42	1,17	1,91	1,43
1,55	1,81	0,86	2,33	2,01	1,48
3,99	3,92	3,71	4,04	2,62	2,77
1,99	2,09	1,36	1,89	2,86	1,81
1,74	2,13	1,46	1,51	1,84	1,1
1,17	1,58	1,48	2,63	1,38	1,46
2,03	2,29	1,54	2,16	2	2,02
0,49	0,43	1,43	1,66	1,41	0,97
0,63	0,49	1,2	1,89	1,53	0,87
1,98	2,2	1,83	1,44	1,89	2,12
0,93	1	1,04	0,63	1,39	0,59
1,09	1,16	1,37	1,39	1,55	1,05
**1,779**	**1,8615**	**1,677**	**2,105**	**1,8415**	**1,974**

Column43	Column44	Column45	Column46	Column47	Column48
A:67(THR)	A:68(GLN)	A:69(ASN)	A:70(ASN)	A:71(PHE)	A:72(PHE)
1,96	4,06	2,92	2,54	1,39	1,2
1,77	2,9	2,19	2,09	1,03	1,09
1,26	3,3	2,06	2,15	2,71	1,13
1,79	3,42	2,05	1,82	1,2	1,12
1,85	2,89	2,32	2,07	1,09	0,69
1,88	3,65	2,14	1,64	1,51	1,21
1,37	3,33	1,59	1,41	1,17	0,91
1,58	3,68	1,78	1,91	1,43	1,26
0,63	2,85	1,3	1,52	1,68	1,08
0,61	2,73	0,86	0,9	1,56	1,01
2,01	3,76	1,84	2,4	2,25	1,49
1,61	2,74	1,32	1,78	1,55	0,56
1,36	2,64	1,51	1,71	0,9	0,6
1,42	3,02	1,34	1,84	0,64	0,59
1,58	3,25	1,58	2,09	1,58	1,12
1,25	2,76	0,64	1,32	0,78	1,1
0,89	2,65	1,07	1,47	0,99	0,93
1,27	2,44	0,86	1,52	1,3	1,03
0,72	2,7	1,04	1,37	0,85	1,08
0,61	2,89	0,81	0,76	0,68	0,8
**1,371**	**3,083**	**1,561**	**1,7155**	**1,3145**	**1**

Column49	Column50	Column51	Column52	Column53	Column54
A:73(LEU)	A:74(LEU)	A:75(ASN)	A:76(LEU)	A:77(ALA)	A:78(ILE)
2,55	1,47	1,5	1,3	1,5	1,18
2,22	1,2	1,34	1,06	1,23	0,92
2,3	1,87	1,27	1,55	1,73	1,71
2,18	1,24	1,44	1,06	1,17	1,21
2,49	1,36	0,76	1,49	1,67	1,7
2,37	1,21	0,98	1,2	0,97	0,53
2,12	0,79	0,86	0,8	0,94	1,05
2,56	1,18	1,08	1,3	1,33	1,27
2,46	1,56	1,34	1,26	1,42	1,56
2,32	0,96	1,14	1,29	1,53	1,28
2,2	2	1,63	1,14	1,57	1,95
2,26	1,21	0,82	0,9	1,09	1,11
1,77	0,85	1,02	0,79	1,05	1,01
1,58	1,22	0,87	1,09	0,97	1,22
1,94	1,39	1,23	1,45	1,36	1,51
1,26	0,71	0,8	0,96	0,55	0,92
2,13	1,01	1,72	1,11	0,97	1,84
1,67	1,03	1,33	0,73	1	2
1,19	0,31	0,54	0,97	0,46	0,64
1,48	0,48	0,85	0,68	0,71	0,87
**2,0525**	**1,1525**	**1,126**	**1,1065**	**1,161**	**1,274**

Column55	Column56	Column57	Column58	Column59	Column60
A:79(SER)	A:80(ASP)	A:81(PHE)	A:82(LEU)	A:83(VAL)	A:84(GLY)
1,48	1,5	1	1,38	1,52	1,25
0,91	0,85	0,86	0,8	0,5	1,1
1,63	1,36	1,56	1,88	2,23	2,23
1,26	1,11	0,9	1,37	1,74	1,42
1,62	1,68	1,77	2,05	1,85	2,02
0,9	1,19	1,16	1,44	1,78	1,58
0,51	0,54	0,87	0,38	0,86	0,84
0,96	1	0,95	0,96	0,73	0,69
1,14	1,15	0,87	0,45	0,78	0,86
1,19	1,13	0,65	0,42	0,98	0,92
1,45	1,36	1,55	1,51	1,62	1,84
0,93	1,38	1,18	1,15	0,89	0,7
0,61	0,77	0,59	1,04	1,17	1,05
1,28	0,84	1,19	1,2	0,63	0,55
1,45	1,46	1,81	1,62	1,67	1,39
0,81	0,34	0,4	0,32	0,34	0,41
0,78	0,82	0,58	0,93	1,23	1,17
0,87	0,68	0,97	1,18	0,85	0,62
0,33	0,28	0,5	0,45	0,4	0,4
0,69	0,54	0,87	0,71	0,45	0,48
**1,04**	**0,999**	**1,0115**	**1,062**	**1,111**	**1,076**

Column61	Column62	Column63	Column64	Column65	Column66
A:85(ALA)	A:86(PHE)	A:87(CYS)	A:88(ILE)	A:89(PRO)	A:90(LEU)
1,18	1,42	1,05	1,19	0,92	0,84
1,18	1,15	0,76	0,84	0,99	1,35
1,77	1,84	1,87	1,82	1,75	2,36
1,16	1,82	1,05	0,94	1,18	1,22
1,75	1,69	1,8	1,99	2,26	2,08
1,6	1,65	1,55	1,71	1,61	1,8
0,68	1,52	0,69	0,82	0,79	0,65
0,82	0,83	0,97	1,13	1,35	1,58
0,74	0,34	0,44	0,35	0,46	0,58
0,7	0,95	0,75	0,87	0,69	1,1
2,59	2,27	1,37	1,76	1,97	2,18
0,84	1,09	1,1	1	1,14	1,46
1,05	1,2	1,01	1,08	0,85	1,3
0,66	0,72	0,72	0,84	0,93	1,21
1,7	1,42	0,95	0,84	0,78	1,42
0,86	0,79	0,6	0,52	0,54	1,03
0,81	1,03	1,31	1,74	1,8	2,2
0,87	0,79	1,1	0,78	0,63	1,22
0,4	0,39	0,58	0,78	0,65	1,93
0,42	0,72	0,52	0,82	0,9	1,18
**1,089**	**1,1815**	**1,0095**	**1,091**	**1,1095**	**1,4345**

Column67	Column68	Column69	Column70	Column71	Column72
A:91(TYR)	A:92(VAL)	A:93(PRO)	A:94(TYR)	A:95(VAL)	A:96(LEU)
1,29	1,39	1,85	1,92	2,36	2,83
0,94	1,56	1,42	0,47	1,62	2,35
2,08	2,41	2,04	1,77	1,51	2,39
1,19	2,1	2,18	1,71	2,33	3,11
1,91	2,24	2,77	2,81	2,24	2,23
2,51	2,52	3,04	2,98	2,43	3,36
1,6	1,29	1,45	1,53	1,6	1,59
1,9	1,66	2,21	1,92	1,42	1,63
0,42	0,55	0,7	0,66	0,51	0,79
1,21	0,69	0,65	0,85	1	0,91
2,09	2,55	2,02	1,9	2,43	3,45
1,23	1,16	0,75	0,69	1,94	1,25
1,57	1,49	0,82	0,78	1,03	1,07
0,88	1,25	1,16	0,58	0,88	0,73
0,82	0,89	0,71	0,65	1,13	1,26
1,15	0,93	0,48	0,75	1,41	1,48
1,83	1,93	2,02	1,51	1,82	1,57
1,33	1,18	1,25	0,65	1,38	1,07
0,91	1,1	0,79	0,63	0,89	1
0,78	0,91	0,99	0,5	1,14	0,84
**1,382**	**1,49**	**1,465**	**1,263**	**1,5535**	**1,7455**

Column73	Column74	Column75	Column76	Column77	Column78
A:97(THR)	A:98(GLY)	A:99(ARG)	A:100(TRP)	A:101(THR)	A:102(PHE)
3,56	2,56	2,42	1	2,23	1,2
2,01	1,21	1,81	1,18	2,3	1,89
2,52	2,05	3,49	1,91	3,17	2,56
3,61	2,54	1,81	1,15	2,26	1,83
2,35	2,83	3,21	2,58	1,84	2,33
3,8	3,88	2,76	1,99	1,55	1,67
2,85	2,3	1,4	0,89	1,04	1,08
2,41	2,03	1,69	1,54	1,09	1,64
0,41	0,45	2,24	0,57	0,59	0,98
1,09	1,46	2,05	0,81	1,41	1,14
3,53	2,9	3,17	1,49	1,69	1,9
0,82	0,63	0,98	0,69	0,85	1,01
0,67	0,74	2,05	1,15	1,23	1,36
0,42	0,41	1,65	0,53	1,03	1,45
0,7	0,84	1,61	0,63	0,63	1,52
1,26	1,34	1,06	0,79	0,96	1,01
0,92	0,9	1,92	1,33	1,14	2,42
1,12	1,12	2,76	1,04	0,96	1,89
0,72	0,8	1,09	0,84	1,01	1
0,67	0,36	0,65	0,5	0,74	1,86
**1,772**	**1,5675**	**1,991**	**1,1305**	**1,386**	**1,587**

Column79	Column80	Column81	Column82	Column83	Column84
A:103(GLY)	A:104(ARG)	A:105(GLY)	A:106(LEU)	A:107(CYS)	A:108(LYS)
0,97	3,32	0,67	0,66	0,76	1,69
1,82	2,82	1,27	1,13	1,25	1,81
1,86	3,36	1,88	1,43	1,38	2,48
1,55	2,86	0,88	0,54	0,65	1,27
2,34	2,88	2,86	2,41	2,55	2,8
1,53	2,76	1,66	1,76	1,53	1,98
1,19	1,77	1	0,86	1,02	1,39
1,4	2,04	1,67	1,75	1,34	1,56
0,86	1,35	0,9	0,97	0,51	0,69
1,08	1,53	0,68	0,93	0,48	0,61
1,33	2,93	1,21	1,05	0,91	1,46
1,02	1,23	1,19	0,83	0,81	1,04
1,03	1,39	0,67	0,57	0,89	1,05
0,88	1,26	1,22	0,57	0,41	1,44
1,03	0,89	1,22	1,06	0,69	0,8
1,2	1,13	1,56	0,94	0,81	1,33
1,37	1,07	1,42	1,87	1,24	1,58
0,81	0,64	0,96	0,76	1,01	1,26
0,88	0,66	1,13	0,72	0,87	1,11
0,92	1,22	1,51	1,41	0,82	0,78
**1,2535**	**1,8555**	**1,278**	**1,111**	**0,9965**	**1,4065**

Column85	Column86	Column87	Column88	Column89	Column90
A:109(LEU)	A:110(TRP)	A:111(LEU)	A:112(VAL)	A:113(VAL)	A:114(ASP)
1,04	0,78	0,47	1,35	0,93	1,47
1,51	0,82	1,69	2,18	1,38	0,95
1,46	1,99	1,65	1,46	1,35	1,41
1,12	0,68	0,44	1,33	1,08	0,96
2,58	2,15	1,81	1,93	1,42	1,23
1,63	1,43	1,59	1,5	1,11	0,95
1,2	0,72	0,79	0,87	0,39	0,8
1,96	1,06	1,06	1,04	0,62	0,61
1,24	0,32	0,62	0,93	0,44	1,51
1,09	0,73	0,79	1,05	0,71	1,56
1,11	1,13	0,84	1,01	0,34	0,29
1,61	1,08	1,04	1,12	0,93	1,62
1	1,33	1,34	1,05	0,81	2,15
1,38	0,68	0,62	1,27	0,93	0,95
1,84	0,92	0,77	0,99	0,75	0,85
1,59	1,14	0,95	1,36	0,67	0,69
2,23	2,08	1,71	2,17	1,72	1,37
1,29	1,29	1,17	1,3	0,84	0,75
1,55	1,47	1,48	1,22	0,95	0,63
1,88	1,08	1,03	1,43	1,18	0,74
**1,5155**	**1,144**	**1,093**	**1,328**	**0,9275**	**1,0745**

Column91	Column92	Column93	Column94	Column95	Column96
A:115(TYR)	A:116(LEU)	A:117(LEU)	A:118(CYS)	A:119(THR)	A:120(SER)
1,3	1,02	0,94	1,44	1,2	0,39
1,96	1,58	1,04	1,43	1,8	1,08
2,32	1,93	1,56	0,95	1,62	1,8
1,43	1	1,2	0,42	1,18	0,62
1,93	1,43	1,91	0,93	1,59	1,21
2,2	1,31	0,92	1,27	1,46	0,51
0,77	0,89	0,56	1,14	1,14	0,63
0,45	0,49	1,14	0,88	0,77	0,68
1,69	1,21	1,2	1,13	0,94	0,65
1,1	1,43	1,6	1,07	1,04	1,06
0,75	0,78	0,7	0,45	1,12	0,57
1,89	1,23	0,94	0,8	0,62	0,39
2,14	0,87	0,56	1,09	0,64	0,61
1,51	1,09	0,9	0,65	1,1	1,35
1,38	0,9	1,19	0,89	0,75	1,16
1,46	1,18	0,9	0,6	0,61	0,65
1,36	1,61	1,85	0,87	1,16	1,26
1,16	1,25	1,2	0,71	0,37	0,62
0,65	1,19	0,58	0,33	0,5	0,48
0,53	1,4	0,92	0,87	0,79	0,63
**1,399**	**1,1895**	**1,0905**	**0,896**	**1,02**	**0,8175**

Column97	Column98	Column99	Column100	Column101	Column102
A:121(LYS)	A:122(ALA)	A:123(PHE)	A:124(ASN)	A:125(ILE)	A:126(VAL)
0,64	0,38	0,82	0,34	0,38	0,44
0,99	1,33	1,5	0,57	1,41	1,08
1,36	1,32	1,46	1,08	1,57	1,39
0,51	0,49	1,05	0,4	0,82	0,81
1,15	1,26	1,17	0,64	1,32	1,04
0,66	0,22	0,49	0,48	0,82	0,91
0,54	0,37	0,92	0,68	0,89	0,79
0,85	0,72	0,98	0,98	1,24	0,98
1,09	1,11	0,65	0,56	1,17	1,1
0,94	1,04	0,86	0,48	1,04	0,97
0,67	1,39	1,74	0,52	1,01	1,01
0,81	0,72	0,75	0,53	1,32	1,41
0,7	0,58	0,58	0,69	1,71	1,25
1,49	1,21	0,92	0,83	1,31	1,02
1,59	1,52	1,37	1	1,59	1,31
0,58	0,82	0,7	0,68	1,3	1,08
1,21	1,27	1,44	0,85	1,52	1,35
0,69	0,94	0,76	0,62	1,79	1,46
0,34	0,53	0,89	0,98	1,33	1,16
0,92	1,05	0,85	0,37	0,99	0,67
**0,8865**	**0,9135**	**0,995**	**0,664**	**1,2265**	**1,0615**

Column103	Column104	Column105	Column106	Column107	Column108
A:127(LEU)	A:128(ILE)	A:129(SER)	A:130(TYR)	A:131(ASP)	A:132(ARG)
1,5	1,13	0,78	1,48	1,81	1,26
0,86	1,66	1,44	1,48	1,41	1,12
1,11	1,99	1,63	1,89	1,73	1,36
0,64	1,02	1,15	1,03	1,01	1,02
0,98	1,79	1,58	1,5	2	1,93
1,03	1,28	1,4	1,47	1,46	1,7
0,8	1,07	1,34	1,09	1,28	1,19
0,74	1,31	1,95	1,56	1,63	1,41
0,64	1,18	1,55	1,16	0,7	1,82
0,51	1,05	1,1	2,06	0,98	1,76
1	1,62	1,12	0,88	1,83	1,33
1,12	1,28	1,47	1,81	1,61	1,05
1,06	1,33	1,96	1,71	0,9	1,73
0,96	1,56	1,94	1,22	1,41	1,36
1,3	1,91	2,3	1,35	1,46	1,85
0,69	1,05	1,67	1	0,97	2,09
0,8	1,56	2,12	1,44	1,32	1,39
1,45	1,97	1,92	1,53	1,36	1,45
1,16	1,89	1,49	1,42	1,53	1,55
1,09	1,26	1,09	1,11	1,04	0,78
**0,972**	**1,4455**	**1,55**	**1,4095**	**1,372**	**1,4575**

Column109	Column110	Column111	Column112	Column113	Column114
A:133(PHE)	A:134(LEU)	A:135(SER)	A:136(VAL)	A:137(THR)	A:138(ARG)
1,01	1,49	1,69	1,52	1,65	1,88
1,81	1,59	2,21	3,22	2,26	2,06
2,15	1,58	1,63	2,29	2,44	2,16
1,78	1,12	1,58	2,89	2,13	1,3
2,38	2,1	2,52	3,19	3,12	3,8
1,77	1,5	1,56	2,37	2,31	3,56
1,14	0,65	0,53	0,5	0,69	1,77
1,43	1,28	1,08	1,5	1,19	1,43
1,46	0,59	0,69	0,86	0,84	1,89
1,9	1,46	1,17	1,28	1,57	3,17
1,34	1,35	1,59	1,32	1,4	1,67
1,83	1,51	1,34	1,57	1,63	4,1
1,65	1,02	0,57	0,73	1,05	1,59
1,39	1,09	1,1	1,2	1,39	2,64
2,06	1,38	1,02	1,72	2,18	3,09
1,83	1,07	0,75	1,22	1,67	1,99
2,38	1,74	1,66	2,24	3,07	3,58
2,25	1,62	1,39	1,66	1,84	2,75
1,93	1,52	1,03	1,28	2,3	3,22
1,45	1,35	1,26	1,38	1,56	3,11
**1,747**	**1,3505**	**1,3185**	**1,697**	**1,8145**	**2,538**

Column115	Column116	Column117	Column118	Column119	Column120
A:139(ALA)	A:140(VAL)	A:141(SER)	A:142(TYR)	A:143(ARG)	A:144(ALA)
1,91	3,15	2,25	0,96	1,5	1,06
2,5	3,79	2,08	1,91	2,79	2,28
1,77	2,16	1,08	1,58	5,57	3,07
1,22	2,28	1,26	1,88	3,61	1,79
2,41	3,52	2,92	2	2,62	2,07
1,59	2,13	1,79	1,71	2,03	1,35
0,35	1,06	1,55	0,96	1,56	0,81
1,1	1,19	1,1	1,43	4,08	0,95
1,55	1,85	2,3	1,42	4,2	3,14
1,7	3,12	2,97	1,61	3,28	3,51
4,3	2,99	2,09	2,38	5,56	2,33
1,63	1,41	1,98	1,38	2,78	2,39
1,2	2,4	2,34	0,91	3,22	2,39
1,32	1,6	0,91	0,97	3	1
1,23	2,37	1,79	1,34	3,23	1,93
1,02	2,37	1,68	1	2,66	2
2,56	3,82	3,87	1,74	3,05	2,46
1,37	2,8	1,93	1,37	3,15	2
2,12	2,85	1,98	1,39	2,54	1,57
1,25	1,72	2,15	1,07	5,38	1,63
**1,705**	**2,429**	**2,001**	**1,4505**	**3,2905**	**1,9865**

Column121	Column122	Column123	Column124	Column125	Column126
A:145(GLN)	A:146(GLN)	A:147(GLY)	A:148(ASP)	A:149(THR)	A:150(ARG)
2,45	1,39	1,01	1,6	1,41	1,72
2,46	3,87	2,17	1,7	1	2,35
1,61	2,23	2,47	2,84	1,23	3,48
1,57	3,63	2,1	1,7	1,02	1,97
1,9	2,15	1,97	1,61	1,08	2,31
2,49	1,4	1,72	2,6	3,7	1,88
3,11	2,01	2,58	1,67	0,99	1,34
3,05	2,17	1,79	1,38	1,03	0,86
3,99	2,02	2,54	1,51	1,41	1,75
5,04	1,88	2,63	1,24	1,06	1,06
2,58	2,82	2,86	3,34	1,91	2,77
3,22	2,49	3,52	1,61	0,94	0,78
4,9	1,35	2,51	1,69	1,14	1,49
2,67	1,6	2,23	1,92	0,67	1,93
2,5	1,92	2,45	2,35	1,63	2,05
4,19	1,56	2,3	1,44	1,35	1,59
3,77	1,42	1,87	1,34	0,9	0,91
3,25	1,43	2,52	1,12	0,97	2,07
2,1	1,62	2,53	1,53	2	3,15
2,84	1,79	1,98	1,63	0,91	0,76
**2,9845**	**2,0375**	**2,2875**	**1,791**	**1,3175**	**1,811**

Column127	Column128	Column129	Column130	Column131	Column132
A:151(ARG)	A:152(ALA)	A:153(VAL)	A:154(ARG)	A:155(LYS)	A:156(MET)
4,86	1,74	2,07	2,49	2,18	2,01
4,89	1,33	1,19	2,34	0,99	1,87
6,49	1,44	1,89	2,64	1,59	2,01
5,55	1,13	1,65	2,35	1,7	1,64
1,31	1,79	1,63	1,63	1,12	1
1,68	1,84	2,34	1,56	1,26	1,56
4,89	1,47	1,21	0,6	0,74	1,07
4,08	1,42	1,28	0,82	0,8	1,36
5,27	1,28	0,71	2,43	1,03	2,02
5,81	1,06	1,04	1,66	0,43	0,65
6,21	1,55	2,4	3,92	1,86	2,03
4,44	1,22	1,29	1,13	0,96	1,2
3,8	0,84	1	0,79	0,79	1,21
4,86	1,88	1,46	1,25	1,06	0,83
4,16	1,24	1,33	1,73	1,08	1,1
3,86	2,43	2,13	2,76	1,83	1,57
3,83	0,96	1,35	0,97	1,5	1,6
4,86	1,27	1,38	1,67	1,28	1,19
3,14	2,18	2,34	1,24	1,45	0,88
2,54	1,1	1,33	1,55	0,99	0,74
**4,3265**	**1,4585**	**1,551**	**1,7765**	**1,232**	**1,377**

Column133	Column134	Column135	Column136	Column137	Column138
A:157(LEU)	A:158(LEU)	A:159(VAL)	A:160(TRP)	A:161(VAL)	A:162(LEU)
2,89	1,67	0,99	1,67	1,4	1,61
1,83	1,63	1,25	2,21	3,34	3,42
1,52	1,79	1,53	1,36	1,7	1,66
1,35	1,34	0,75	1,95	2,07	2,45
1,76	0,85	0,67	1,35	1,54	1,62
1,24	0,72	0,27	0,8	1,18	0,85
1,5	0,88	0,96	1,19	1,56	1,02
1,41	1,08	0,82	1,42	1,08	0,88
1,12	1,28	1,03	1,06	0,97	0,74
1,05	0,94	0,53	1,51	0,98	1,58
2,55	2,39	1,14	1,95	2,37	2,83
0,95	1,51	0,68	1,49	0,79	1,02
1,73	0,86	0,86	0,8	0,94	0,92
1,7	0,86	0,7	1,64	1,5	1,37
1,59	0,83	0,93	1,05	1,19	1,29
1,17	1,2	0,38	1,24	1,06	1,1
2,15	2,51	1,23	2,43	2,21	2,2
1,17	0,37	0,29	1,66	0,62	1,27
1,33	0,34	0,28	0,62	0,59	1,38
1,03	0,64	0,56	1,18	1,1	1,69
**1,552**	**1,1845**	**0,7925**	**1,429**	**1,4095**	**1,545**

Column139	Column140	Column141	Column142	Column143	Column144
A:163(ALA)	A:164(PHE)	A:165(LEU)	A:166(LEU)	A:167(TYR)	A:168(GLY)
0,87	2,13	1,65	2,18	2,19	2,05
2,05	3,55	3,35	3,45	3,09	3,4
1,31	1,73	1,94	2,81	2,34	1,98
1,44	3,47	3,03	2,57	2,6	2,63
1,22	1,84	2,17	2,29	2,46	2,36
1,06	2,27	1,8	1,85	2,34	2,34
0,88	2,58	2,31	2,07	2,69	2,26
0,69	1,15	2,32	1,26	1,34	1,06
1,17	1,41	1,75	0,66	1,04	0,79
0,59	1,38	1,27	1,2	0,87	0,95
1,32	2,48	2,54	2,16	1,79	1,8
0,61	0,98	1,69	1,18	1,07	1,07
0,76	0,76	1,44	1,21	1,28	1,49
0,89	1,39	1,51	1,56	1,39	1,84
0,68	1,02	1,38	0,99	0,95	0,72
0,32	1,37	2,04	1,85	1,24	1,47
1,72	2,57	2,92	2,68	1,89	2,22
0,45	1,16	2,1	1,8	1,72	1,69
0,32	1,77	2,14	1,38	1	1,44
0,53	0,95	1,85	1,64	0,98	0,97
**0,944**	**1,798**	**2,06**	**1,8395**	**1,7135**	**1,7265**

Column145	Column146	Column147	Column148	Column149	Column150
A:169(PRO)	A:170(ALA)	A:171(ILE)	A:172(LEU)	A:173(SER)	A:174(TRP)
3,18	2,71	1,99	2,44	2,93	3,02
5,15	4,57	2,98	3,69	4,57	4,22
3,66	2,72	1,53	2,95	5,12	3,33
4,04	3,45	2,09	2,67	3,82	3,26
3,49	3,76	3,1	3,1	3,39	4,05
2,94	2,92	2,29	2,46	2,54	3,35
2,99	2,81	1,87	2,15	3,45	3,46
2,7	2,49	1,56	1,65	2,35	2,67
1,47	1,2	0,83	0,63	1,39	0,91
1,34	1,59	0,94	1,19	2,03	1,53
2,85	3,1	2,03	2,07	2,92	3,55
1,9	1,56	0,99	1,29	1,97	1,48
1,98	1,33	1,14	1,28	2	0,91
2,13	2,29	1,88	2,15	2,56	1,93
1,13	1,17	0,92	0,86	1,64	0,61
1,75	1,85	1,26	1,55	2,17	1,43
2,25	2,72	2,78	2,83	4,03	2,91
2,52	2,48	1,56	1,68	2,67	1,83
2,26	2,1	1,92	2,3	2,42	1,79
1,33	1,01	1,24	1,1	1,06	0,73
**2,553**	**2,3915**	**1,745**	**2,002**	**2,7515**	**2,3485**

Column151	Column152	Column153	Column154	Column155	Column156
A:175(GLU)	A:176(TYR)	A:177(LEU)	A:178(SER)	A:179(GLY)	A:180(GLY)
3,42	5,24	4,04	4,73	4,23	3,36
4,67	6,4	4,35	6,17	4,53	4,83
4,28	7,24	4,1	6,52	4,73	5,11
3,59	5,23	4,18	5,87	4,67	4,39
5,38	6,07	4,64	6,55	4,22	4,83
4,05	4,73	4,01	5,43	3,56	4,69
5,54	8,03	4,77	6,44	5,32	5,62
3,98	5,96	2,74	4,22	2,94	4,39
2,27	3,19	1,54	2,77	1,51	1,63
2,95	3,6	1,89	3,68	2,61	3,51
3,98	6,85	5,91	6,97	4,92	3,8
1,6	4,32	2,09	3,03	1,98	2,2
1,65	2,44	1,9	2,73	2,74	2,33
2,79	2,98	1,06	2,86	2,21	3,76
1,55	3,69	1,33	1,69	2,35	2,2
2,44	2,6	1,01	2,92	2,98	4,56
4,18	5,94	2,96	4,9	3,55	4,69
3,11	3,77	1,82	3,38	2,34	2,86
2,84	3,25	1,84	2,93	2,33	2,76
1,26	1,27	1,51	1,68	2,91	1,79
**3,2765**	**4,64**	**2,8845**	**4,2735**	**3,3315**	**3,6655**

Column157	Column158	Column159	Column160	Column161	Column162
A:181(SER)	A:182(SER)	A:183(ILE)	A:184(PRO)	A:185(GLU)	A:186(GLY)
2,33	4,54	2,63	2,2	2,97	0,58
3,21	3,47	2,76	1,47	2,09	1,14
4,08	3,88	3,27	3,18	2,27	1,57
2,88	3,7	2,06	1,55	1,66	0,49
4,11	4,89	3,89	3,21	2,67	2,14
3,07	4,26	3,61	3,82	3,51	2,76
4,25	5,69	4,03	2,75	3,04	1,02
3,23	4,7	3,13	3,32	2,94	1,48
0,57	1,19	1,11	1,74	3,61	0,84
2,3	1,71	1,04	2,01	2,62	0,94
2,43	3,34	2,49	2,46	2,99	1,6
1,26	1,01	0,99	2,61	3,21	1,26
1,99	1,17	0,83	0,81	3,27	1,51
2,55	1,89	0,85	0,98	2,66	0,88
1,72	0,87	0,27	0,3	2,83	0,58
3,24	1,81	1,2	0,89	1,72	1,21
2,95	2,74	1,28	0,54	1,65	0,32
1,93	1,32	0,93	1,21	2,06	1,17
1,43	0,87	0,61	0,58	1,86	1,22
1,49	1,14	0,87	0,97	2,17	0,69
**2,551**	**2,7095**	**1,8925**	**1,83**	**2,59**	**1,17**

Column163	Column164	Column165	Column166	Column167	Column168
A:187(HIS)	A:188(CYS)	A:189(TYR)	A:190(ALA)	A:191(GLU)	A:192(PHE)
0,79	1,04	2,9	1,8	2,29	2,01
1,67	1,14	2,89	2,76	3,13	3,22
2,54	1,93	3,9	2,51	2,52	2,1
0,54	0,7	2,87	2,5	2,65	2,5
2,88	2,86	4,21	3,65	3,97	2,97
3,55	2,41	3,52	2,58	3,07	2,15
2,21	1,7	2,47	2,45	3,08	1,48
2,78	1,89	1,94	2,51	2,7	1,32
1,17	0,64	1,64	1,31	1,11	1,2
0,9	0,9	1,2	1,17	1,44	0,6
1,78	1,3	3,13	2,73	2,86	2,28
0,91	0,72	2,24	1,52	1,27	1,31
0,95	1	1,91	1,05	0,4	1,58
0,96	0,47	1,5	1,04	1,31	1,21
0,49	0,32	1,92	0,28	0,29	0,54
0,54	0,78	1,79	0,99	1,22	1,49
0,55	0,95	1,58	1,85	2	1,74
0,58	0,41	1,18	1,63	1,43	1,42
0,81	0,67	0,66	1,11	1,03	1,67
0,76	0,53	0,62	1,2	0,79	0,93
**1,368**	**1,118**	**2,2035**	**1,832**	**1,928**	**1,686**

Column169	Column170	Column171	Column172	Column173	Column174
A:193(PHE)	A:194(TYR)	A:195(ASN)	A:196(TRP)	A:197(TYR)	A:198(PHE)
3,08	3,67	3,71	4,79	5,86	3,22
3,46	4,17	4,56	5,02	6,32	4,56
3,83	4,51	4,18	5,04	6,97	4,08
3,35	4,26	3,66	4,33	5,42	3,49
4,21	5,14	4,25	4,51	4,95	3,54
3,4	4,27	3,72	4,44	4,61	3,11
2,38	3,35	3,24	3,11	3,82	2,12
2,05	2,53	1,95	2,21	1,69	2,21
1,08	1,27	0,71	1,9	1,48	1,08
1,23	1,57	1,28	2,19	2,29	1,43
3,91	4,48	4,3	5,1	5,37	3,19
1,67	2,26	1,62	2,71	3,09	2,13
1,13	1,26	1,06	2,29	1,41	1,02
1,35	1,98	2,04	2,7	2,95	1,83
1,77	1,54	1,09	2,43	2,32	1,02
1,58	1,99	1,7	2,36	3,87	1,73
2,03	3,26	2,9	2,92	4,62	3,09
1,31	2,34	1,65	1,81	2,59	1,76
0,89	1,58	1,7	1,7	2,11	2,05
0,53	0,9	0,72	0,64	2,25	0,91
**2,212**	**2,8165**	**2,502**	**3,11**	**3,6995**	**2,3785**

Column175	Column176	Column177	Column178	Column179	Column180
A:199(LEU)	A:200(ILE)	A:201(THR)	A:202(ALA)	A:203(SER)	A:204(THR)
3,11	2,8	3,25	2,05	2,4	1,97
4,09	5,02	5,12	3,33	3,7	3,95
3,97	4,51	4,6	2,67	3,35	3,32
3,21	3,8	4,04	2,37	2,57	2,66
3,75	3,51	3,49	2,04	2,79	2,26
2,75	2,62	2,41	1,45	2,11	1,3
1,93	2,43	2,09	1,39	1,69	1,12
2,15	2,1	1,77	0,7	1,06	0,99
0,74	2,05	1,82	1,13	1,05	1,26
1,2	1,53	0,89	0,83	1,06	1,38
3,57	3,85	4,01	2,41	2,74	2,85
0,99	1,46	1,76	0,87	0,79	1,02
1,19	2,34	1,53	0,73	0,6	0,58
1,39	2,03	1,01	1,08	1,05	0,81
1,25	1,83	1,06	0,77	1,07	1,02
1,63	1,62	1,4	0,6	0,82	0,69
2,62	3,17	2,54	2,28	2,32	2,22
1,31	2,43	1,47	1,01	1,09	1,2
1,33	2,14	1,64	1,51	1,14	1,22
1,01	1,17	1,5	1,26	1,2	1,37
**2,1595**	**2,6205**	**2,37**	**1,524**	**1,73**	**1,6595**

Column181	Column182	Column183	Column184	Column185	Column186
A:205(LEU)	A:206(GLU)	A:207(PHE)	A:208(PHE)	A:209(THR)	A:210(PRO)
1,86	1,69	2,07	1,77	1,27	0,64
4,02	2,54	2,71	3,42	2,05	1,26
3,4	2,26	2,81	3,29	1,96	1,03
2,33	1,56	1,45	3,39	0,58	0,93
2,43	2,08	1,67	1,87	0,65	0,96
1,4	1,12	0,72	0,83	1,15	1,3
1,37	1,17	0,56	0,66	0,93	1,23
1,2	0,8	1,39	1,03	0,69	0,93
1,26	1,37	1,54	1,19	1,58	1,86
1,36	1,14	1,27	1,28	1,32	1,04
2,56	2,17	2,25	2,35	1,77	1,41
1,47	1,46	1,83	1,52	1,35	1,54
0,78	0,64	1,19	1,68	1,41	1,73
1,41	1,18	1,67	0,95	0,91	1,44
1,22	0,91	1,53	1,03	1,01	1,55
1,26	0,97	0,54	0,58	0,59	1,3
1,91	1,68	1,72	2,01	1,74	1,49
0,74	0,92	1,21	1,48	0,92	1,43
1,27	1,09	1,69	2,24	0,78	1,02
1,73	1,21	1,56	1,26	0,63	0,94
**1,749**	**1,398**	**1,569**	**1,6915**	**1,1645**	**1,2515**

Column187	Column188	Column189	Column190	Column191	Column192
A:211(PHE)	A:212(LEU)	A:213(SER)	A:214(VAL)	A:215(THR)	A:216(PHE)
1,88	1,7	1,13	0,87	1,37	2,48
2,69	2,1	1,6	1,28	2,25	3,49
2,98	1,93	1,12	0,94	1,9	3,08
3,57	1,43	1,29	0,97	1,8	3,41
2,39	1,99	1,79	1,16	2,3	4,98
1,54	1,84	1,17	0,96	1,56	4,32
1,5	1,04	1,32	0,87	1,74	3,67
0,64	1,14	1,08	0,81	1,39	3,18
1,41	2,85	1,9	1,49	2,75	4,94
0,77	0,82	1,2	0,78	1,86	3,04
3,21	1,59	1,47	1,27	1,97	3,22
1,7	2,38	2,16	1,16	2,53	5,67
1,91	2,37	2,41	1,98	3,08	2,35
1,93	1,47	1,64	1,42	2,23	4,25
1,53	1,86	1,67	2,33	3,97	2,37
1,25	1,56	1,54	1,49	2,52	1,56
2	1,53	1,28	1,97	3,65	2,65
2,12	1,74	1,92	1,71	2,19	2,5
1,7	1,36	1,42	1,22	1,96	3,31
1,19	0,7	1,15	1	1,4	1,71
**1,8955**	**1,67**	**1,513**	**1,284**	**2,221**	**3,309**

Column193	Column194	Column195	Column196	Column197	Column198
A:217(PHE)	A:218(ASN)	A:219(LEU)	A:220(SER)	A:221(ILE)	A:222(TYR)
1,13	1,87	1,86	1,47	1,24	5,26
0,87	2,8	2,87	1,51	2,33	5,96
1,66	1,8	3,34	1,8	1,58	3,49
1,12	2,07	1,84	1,27	1,51	4,91
2,35	2,29	2,55	1,76	2,09	5,22
1,34	1,46	1,2	1,18	1,14	4,79
1,22	1,52	0,87	1,01	0,79	4,36
1,74	1,31	1,54	1,29	1,12	2,86
1,55	1,41	1,47	0,85	0,69	1,51
1,75	1,76	1,78	1,53	1,25	1,28
1,01	2,11	1,73	1,53	1,3	5,19
2,01	1,98	1,97	1,73	1,88	1,77
2,21	1,87	1,16	0,99	1,13	1,62
1,32	1,51	0,99	0,95	0,89	1,48
1,97	2,22	2,83	2,28	2,03	3,07
1,9	2,19	2,22	1,91	1,64	1,74
1,93	2,03	2,82	2,38	2,17	2,8
2,03	2,41	2,45	2,1	1,93	4,38
1,48	1,75	2,37	1,91	1,54	2,81
1,3	1,79	1,01	1,21	1,6	1,38
**1,5945**	**1,9075**	**1,9435**	**1,533**	**1,4925**	**3,294**

Column199	Column200	Column201	Column202	Column203	Column204
A:223(LEU)	A:224(ASN)	A:225(ILE)	A:226(GLN)	A:227(ARG)	A:228(ARG)
1,6	0,43	1,17	2,7	2,1	1,04
2,23	1,88	2,47	2,55	2,29	2,76
3,54	2,21	2,91	4,32	4,12	2,8
1,19	1,18	0,92	1,22	2,98	2,35
2,58	1,8	1,75	2,12	2,05	1,95
1,12	0,63	1	1,42	2,22	1,12
1,85	0,76	0,71	0,97	1,51	0,96
2,44	1,55	2,28	3,06	4,26	2,27
0,97	0,92	1,1	1,7	2,11	1,48
1,38	1,29	0,5	1,64	2,23	1,31
2,02	1,43	1,57	1,86	2,17	1,49
1,59	1,52	1,03	1,31	3,12	1,26
1,24	1,08	1,46	1,99	2,14	1,51
1,35	1,11	1,57	2,18	2,12	1,48
2,87	2,49	2,37	3,03	4,1	2,84
2,39	1,75	1,58	1,26	1,21	1,78
2,67	2,66	3,04	3,23	3,93	2,86
2,07	1,9	1,55	2,1	1,78	1,8
2,47	1,77	1,76	3,55	3,93	1,26
0,95	1,18	1,46	1,47	0,96	1,98
**1,926**	**1,477**	**1,61**	**2,184**	**2,5665**	**1,815**

Column205	Column206	Column207	Column208	Column209	Column210
A:229(THR)	A:230(ARG)	A:231(LEU)	A:232(ARG)	A:233(LEU)	A:234(ASP)
2,8	4,53	8,69	9,59	16,98	16,65
4	4,23	11,4	9,68	17,66	16,27
5,08	6,17	9,27	10,98	15,07	13,21
1,32	6,05	7,76	8,24	15,12	14,31
1,42	2,46	9	5,94	13,95	11,84
2,29	4,98	6,73	9,45	16,4	16,17
1,23	1,75	8,74	8,55	15,74	15,75
4,37	8,83	7,42	11,33	18,67	18,13
3,02	3,92	8,58	13,22	18,46	19,98
1,77	3,5	7,93	11,53	16,75	17,94
3,25	5,65	8,56	11,99	16,8	18,79
1,99	3,06	8,61	13,12	18,28	20,23
2,85	5,02	8,02	12,89	18,65	20,17
2,92	5,3	7,81	11,33	17,86	18,35
4,19	5,58	10,55	14,32	19,8	21,38
0,65	4,07	7,1	6,66	13,37	12,76
3,08	3,66	9,43	8,41	15,58	15,06
1,07	4,81	7,89	9,12	14,12	15,15
1,57	3,76	6,69	11,18	13,98	17,26
1,62	2,14	7,57	8,49	13,99	14,88
**2,5245**	**4,4735**	**8,3875**	**10,301**	**16,3615**	**16,714**

Column211	Column212	Column213	Column214	Column215	Column216
A:235(GLY)	A:236(ALA)	A:237(ARG)	A:238(GLU)	A:239(ALA)	A:240(ALA)
15,92	13,38	9,71	10,76	9,63	12,32
16,7	14,39	13,55	11,4	13,64	11,17
14,49	13,92	13,23	8,9	9,46	7,91
12,94	11,2	10,04	8,42	12,18	11,48
11,81	11,6	12,23	8,3	11,39	10,06
13,88	9,97	8,35	8,65	7,27	7,85
15,76	13,57	10,54	9,48	11,5	12,54
13,1	9,35	13,55	9,34	10,02	12,14
14,65	9,35	9,46	8,66	9,11	11,15
11,84	6,03	7,71	7,24	8,42	9,14
17,32	13,32	11,14	10,32	15,1	13,88
14,82	9,29	10,14	7,96	9,38	8
15,01	10,66	13	8,55	9,71	11,28
13,68	10,84	16,75	9,13	10,67	11,79
17,17	14,4	18,68	11	12,63	13,61
9,84	8,53	14,78	6,34	5,62	7,46
10,36	10,1	17,02	8,85	7,49	8,67
9,04	8,13	14,15	6,93	4,64	5,94
11,62	8,85	12,83	6,35	4,93	7,36
9,65	8,82	14,56	8,6	8,43	5,47
**13,48**	**10,785**	**12,571**	**8,759**	**9,561**	**9,961**

Column217	Column218	Column219	Column220	Column221	Column222
A:241(GLY)	A:347(ARG)	A:348(PHE)	A:349(ARG)	A:350(LEU)	A:351(SER)
17,04	12,39	5,24	9,33	2,3	3,38
15,01	2,31	4,45	6,3	2,05	2,98
5,93	5,26	10,18	6,16	2,59	2,43
13,73	3,18	1,98	6,46	2,34	3,37
13,1	5,83	3,83	6,81	1,26	1,64
10,61	6,73	3,72	5,19	2,05	2,75
15,24	12,23	3,67	6,17	1,68	3,09
16,12	6,46	4,4	6,37	2,64	2,42
13,54	6,26	4,54	5,23	2,42	2,86
11,86	4,25	3,52	4,49	2,05	1,04
12,5	10,11	5,22	7,07	1,8	1,86
11,38	6	4,11	5,08	2,03	2,31
14,86	7,15	4,44	5,53	2,63	2,46
15,4	4,34	4,56	6,57	2,62	2,25
16,01	10,63	6,1	7,93	2,47	3
9,36	2,3	1,51	3,62	1,56	2,66
13,19	6,62	5,77	7,24	2,58	1,36
11,29	8,47	6,65	6,98	1,35	1,14
12,92	2,35	1,4	3,77	1,43	2,93
10,29	4,54	3,17	4,81	1,85	1,53
**12,969**	**6,3705**	**4,423**	**6,0555**	**2,085**	**2,373**

Column223	Column224	Column225	Column226	Column227	Column228
A:352(ARG)	A:353(ASP)	A:354(ARG)	A:355(LYS)	A:356(VAL)	A:357(ALA)
2,57	0,82	3,5	2,41	2,18	2,18
4,45	2,25	3,28	2,8	1,59	0,75
2,37	1,9	1,41	3,14	2,17	1,35
5,04	1,48	2,59	3,75	2,27	1,17
2,31	1,62	2,67	1,76	1,97	1,18
3,32	0,86	1,77	3,05	2,2	1,7
3,14	0,94	3,36	3,38	1,62	1,79
3,65	1,33	0,94	1,52	1,16	0,96
2,99	1,48	2,38	2,32	1,7	1,47
1,8	2,09	1,04	1,8	0,89	1,7
2,61	1,03	2,07	3,2	1,85	2,04
2,44	1,03	1,53	1,42	0,96	0,84
2,22	1,55	1,69	2,64	1,01	1
2,89	2,02	1,44	0,7	0,6	1,19
2,77	1,66	2,36	4,81	1,18	1,39
1,87	1,42	3,75	2,77	1,87	1,53
2,27	2,09	2,33	2,26	1,19	1,06
1,72	1,2	2,69	2,17	1,07	1,47
4,2	0,63	3,27	2,72	0,83	0,94
3,27	1,9	3,51	2,38	1,33	1,82
**2,895**	**1,465**	**2,379**	**2,55**	**1,482**	**1,3765**

Column229	Column230	Column231	Column232	Column233	Column234
A:358(LYS)	A:359(SER)	A:360(LEU)	A:361(ALA)	A:362(VAL)	A:363(ILE)
2,03	2,76	4,82	2,49	0,86	1,82
3,33	0,75	2,34	1,63	1,67	0,96
2,09	1,79	4,4	1,92	1,8	2,22
2,97	1,95	2,97	2,73	1,27	1,73
1,2	2,66	5,16	2,63	2,46	2,7
2,75	3,07	5,55	2,15	0,94	2,6
3,02	3,41	6,19	2,14	0,95	1,61
2,24	3,04	5,65	1,93	1,2	2,68
2,49	3,17	6,18	3,05	1,7	2,26
3,48	2,69	6,04	2,54	1,26	2,04
2,74	2,48	2,41	1,63	1,76	1,89
1,77	2,92	5,66	2,62	2,19	2,76
1,91	2,97	4,76	2,58	1,87	2,07
3,57	1,58	2,66	2,11	1,59	1,83
3,12	3,45	5,33	2,94	1,76	2,49
1,7	1,92	1,93	1,95	1,72	1,5
3,08	1,8	3,73	1,8	1,19	1,57
2,37	2,08	2,89	1,58	0,95	0,82
2,74	1,84	2,72	1,21	0,98	0,82
4,02	2,29	3,49	1,16	0,85	1,14
**2,631**	**2,431**	**4,244**	**2,1395**	**1,4485**	**1,8755**

Column235	Column236	Column237	Column238	Column239	Column240
A:364(VAL)	A:365(SER)	A:366(ILE)	A:367(PHE)	A:368(GLY)	A:369(LEU)
1,37	2,08	1,64	0,56	1,51	1,66
1,51	2,58	1,88	1,41	2,61	2,69
1,41	1,55	1,8	1,19	2,03	2,38
2,36	2,48	1,49	1,04	1,58	1,57
2,36	1,86	1,56	1,26	1,63	2,04
2,18	1,5	0,69	0,44	0,65	0,93
1,15	1,06	0,84	0,56	0,94	1,23
2,01	1,12	0,8	0,48	0,68	0,76
2,22	1,5	1,39	1,03	1,63	2,38
1,9	1,24	0,58	0,98	0,79	1,55
1,65	2,77	2,19	1,48	2,1	2,07
2,23	1,54	2,11	1,08	1,45	1,82
2,22	1,74	2,26	1,3	1,54	3,06
1,61	1,93	2,24	0,99	1,2	1,95
3,26	2,58	1,87	0,83	0,74	1,61
1,21	1,27	1,15	0,97	0,54	1,18
1,99	1,79	1,12	1,14	1,23	1,9
0,97	1,05	0,51	0,43	0,52	1,16
0,58	1,12	0,6	0,39	0,67	1,79
1,24	1,85	1,48	1,16	1,66	1,28
**1,7715**	**1,7305**	**1,41**	**0,936**	**1,285**	**1,7505**

Column241	Column242	Column243	Column244	Column245	Column246
A:370(CYS)	A:371(TRP)	A:372(ALA)	A:373(PRO)	A:374(TYR)	A:375(THR)
1,47	1,67	1,28	1,48	1,37	1,67
1,69	1,43	1,73	1,85	1,67	2,44
1,56	1,68	1,75	2,05	1,92	1,86
1,05	0,98	1,15	1,32	1,18	1,74
1,43	1,13	1,98	1,98	2,04	2,07
0,99	1,13	0,44	0,75	1,1	1,24
1,01	0,73	0,62	1,05	0,56	0,74
1,32	1,08	1,19	1,8	0,92	1,04
1,92	1,46	1,93	2,39	1,74	1,74
1,33	1,06	1,2	1,98	1,22	1,05
1,61	1,06	1,7	1,84	1,81	1,93
1,53	1,51	1,52	1,64	1,48	1,4
2,06	1,43	1,82	2,28	1,46	1,68
1,52	1,57	1,86	2	1	1,73
0,86	0,84	0,68	1,29	0,73	0,77
0,37	0,61	0,56	0,7	0,58	0,54
1,36	1,17	1,56	1,95	1,7	2,24
0,65	0,94	1,06	1,42	1,1	0,79
1,34	0,9	1,29	1,84	0,87	1,03
0,55	0,91	1,41	1,65	0,67	1,72
**1,281**	**1,1645**	**1,3365**	**1,663**	**1,256**	**1,471**

Column247	Column248	Column249	Column250	Column251	Column252
A:376(LEU)	A:377(LEU)	A:378(MET)	A:379(ILE)	A:380(ILE)	A:381(ARG)
3,51	3,27	2,5	2,91	3,67	4,11
3,92	3,26	2,32	3,34	3,66	3,92
3,27	2,73	2,51	2,85	2,96	4,08
3,93	2,77	1,92	3,02	3,24	3,77
4,17	2,38	2,32	2,33	2,54	3,51
3,3	2,01	1,74	2,22	2,85	3,41
2,25	1,49	1,55	1,66	1,92	2,63
1,6	0,88	0,93	0,99	1,51	1,65
1,54	2,27	2,47	2,19	2,67	3,17
1,52	1,76	1,72	1,87	1,94	2,72
3,43	2,94	2,48	2,82	2,96	4,14
1,38	1,7	1,63	1,94	2,19	3,16
2,31	2,24	2	2,29	2,76	3,34
2,89	2,12	1,43	2,03	2,57	2,79
1,26	1,39	1,29	1,76	2,64	3,42
1,72	1,31	0,91	1,17	2,04	2,89
3,11	1,73	1,42	2,32	2,04	2,06
0,76	1,09	1,18	0,62	0,41	1,02
0,93	1,63	1,71	1,27	2,01	1,2
1,75	1,58	0,88	1,15	1,38	1,72
**2,4275**	**2,0275**	**1,7455**	**2,0375**	**2,398**	**2,9355**

Column253	Column254	Column255	Column256	Column257	Column258
A:382(ALA)	A:383(ALA)	A:384(CYS)	A:385(HIS)	A:386(GLY)	A:387(HIS)
4,98	3,28	2,85	2,77	3,43	8,98
4,31	2,81	3,5	4,57	2,91	7,61
4,87	2,94	3,09	3,4	2,89	3,58
4,49	2,98	3,21	3,93	3,54	4,58
4,14	2,67	3,35	4,17	4,14	3,83
4,13	2,54	2,08	3,36	3,11	2,55
3,24	1,68	1,71	2,43	2,58	2,87
3,43	2,61	0,87	1,23	1,05	1,99
4,25	4,14	2,12	2,21	0,97	2,02
3,64	3,22	1,94	2,06	1,96	2,01
4,95	3,24	3,02	3,65	2,92	7,91
3,95	2,97	1,86	2,68	2,33	2,34
3,93	3,52	2,11	1,94	1,84	2,22
3,23	2,02	0,77	1,45	1,22	1,78
4,02	2,65	1,74	4,33	2	3,14
3,44	2,34	1,57	2,39	1,94	2,35
2,68	1,35	1,18	2,06	2,21	2,53
2,37	2,38	1,38	0,73	0,9	2,05
3,05	2,97	1,49	2,06	0,8	1,36
1,57	1	1,44	1,94	1,21	2,19
**3,7335**	**2,6655**	**2,064**	**2,668**	**2,1975**	**3,3945**

Column259	Column260	Column261	Column262	Column263	Column264
A:388(CYS)	A:389(VAL)	A:390(PRO)	A:391(ASP)	A:392(TYR)	A:393(TRP)
4,26	4,2	3,68	2,29	3,69	5,51
4,01	4,02	3,46	1,8	3,54	5,94
3,57	3,4	3,32	2,82	3,7	3,28
3,61	2,69	3,06	2,73	4,33	3,09
3,01	3,5	3,33	2,6	2,77	2,99
1,78	2,69	2,12	1,8	1,82	1,94
1,78	2,88	2,19	1,54	1,27	2,93
1,14	1,54	1,02	1,02	1,87	2,39
2,06	2,08	2,22	2,08	2,94	2,34
1,9	2,07	1,87	1,92	2,56	2,19
3,8	4,02	3,78	2,67	3	3,37
1,23	1,43	1,38	0,83	1,66	1,65
1,67	1,73	1,12	1,79	1,84	1,27
1,52	2,1	1,39	1,44	0,67	0,83
1,05	2,23	1,93	1,61	0,84	1,08
0,81	1,67	1,21	0,41	1	1,07
1,57	3,04	2,45	1,74	1,06	1,52
1,43	1,45	1,12	0,75	0,63	1,7
1,3	1,84	1,75	1,85	2,53	2,22
1,33	1,32	1,01	1,34	1,8	0,65
**2,1415**	**2,495**	**2,1705**	**1,7515**	**2,176**	**2,398**

Column265	Column266	Column267	Column268	Column269	Column270
A:394(TYR)	A:395(GLU)	A:396(THR)	A:397(SER)	A:398(PHE)	A:399(TRP)
2,46	2,04	2,08	1,4	0,84	1,74
2,29	1,83	1,84	1,54	1,06	1,44
3,87	1,57	2,16	1,87	1,78	1,32
2,87	1,97	1,66	1,17	0,6	1,57
2,62	2,75	2,75	3,16	3,02	1,39
1,84	1,48	1,48	1,71	2,81	2,23
1,33	1,88	1,08	1,81	1,64	1,46
0,61	1,51	1,24	1,07	1,71	1,58
1,81	1,89	2,26	1,47	2,73	1,57
1,56	1,85	2	1,09	2,33	1,64
2,73	2,18	2,07	1,85	1,39	1,53
1,21	1,55	1,24	1,7	1,78	1,12
1,56	1,93	1,4	1,72	1,81	1,31
1,27	1,26	1,25	1,65	1,27	1,36
1,25	1,44	1,11	1,76	1,09	0,83
0,85	1,02	0,66	1,05	0,85	0,38
1,67	1,36	1,24	2,18	1,97	1,11
0,51	1,02	0,68	1,37	1,25	0,94
1,16	1,3	1,01	1,57	1,07	1,38
0,39	1,02	0,54	1,21	1,08	0,46
**1,693**	**1,6425**	**1,4875**	**1,6175**	**1,604**	**1,318**

Column271	Column272	Column273	Column274	Column275	Column276
A:400(LEU)	A:401(LEU)	A:402(TRP)	A:403(ALA)	A:404(ASN)	A:405(SER)
2,41	1,14	1,64	2,12	1,36	1,49
2,07	0,74	0,83	1,15	0,91	0,75
2,11	1,93	1,43	1,9	1,5	1,5
1,89	0,73	1,4	1,5	0,94	1,12
2,41	1,36	2,13	1,68	1,39	1,7
1,51	1,04	1,71	0,73	0,67	0,97
0,98	0,83	1,3	0,57	0,53	0,53
0,44	0,59	1,27	0,4	0,97	0,54
1,19	1,22	1,38	1,25	1,58	1,13
1,29	1,03	1,36	1,04	1,13	0,86
2,02	0,9	0,71	2,09	1,51	1,47
1,15	1,51	1,25	1,35	1,3	1,17
0,97	1,36	1,19	1,04	1,7	0,84
0,73	0,88	0,82	0,57	1,54	0,58
1,08	1,21	0,94	0,82	1,7	1,43
0,53	0,7	1,05	0,71	0,75	0,32
0,75	1,17	1,13	0,59	1,16	0,82
0,55	0,61	0,77	0,56	0,45	0,39
0,42	0,73	0,83	0,67	0,53	0,41
0,74	0,77	0,41	0,27	0,51	0,32
**1,262**	**1,0225**	**1,1775**	**1,0505**	**1,1065**	**0,917**

Column277	Column278	Column279	Column280	Column281	Column282
A:406(ALA)	A:407(VAL)	A:408(ASN)	A:409(PRO)	A:410(VAL)	A:411(LEU)
1,92	1,8	1,93	1,69	1,75	1,77
1,48	1,77	1,12	1,1	1,26	1,05
2,25	1,96	1,89	1,67	2,19	1,89
1,62	1,28	1,14	1,28	1,18	1,34
2,24	1,96	1,48	1,85	2,05	2,66
1,06	1,12	1,17	1,16	1,96	1,91
1,53	1,24	1,7	2,35	1,88	1,75
0,79	1,26	1,76	1,95	1,76	1,74
1,31	1,79	1,76	1,27	1,5	1,51
0,85	1,25	1,32	1,35	1,26	1,23
2,05	1,84	1,42	1,7	2,2	1,13
1,51	1,66	1,3	1,4	1,91	2,63
0,79	0,94	0,72	0,75	0,79	1,36
0,54	0,83	0,68	0,5	0,82	0,55
1,67	1,91	1,54	1,85	1,79	2,12
0,62	0,47	0,45	0,48	0,63	0,47
0,9	0,67	1,23	1,26	0,77	0,68
0,49	0,71	0,62	0,99	1,34	1,13
0,49	0,68	0,39	0,75	1,03	1,09
0,72	0,88	0,72	0,82	1,06	1,16
**1,2415**	**1,301**	**1,217**	**1,3085**	**1,4565**	**1,4585**

Column283	Column284	Column285	Column286	Column287	Column288
A:412(TYR)	A:413(PRO)	A:414(LEU)	A:415(CYS)	A:416(HIS)	A:417(HIS)
1,8	1,84	1,77	2,38	2,42	3,8
1,25	0,72	1,72	1,53	1,55	4,87
1,82	1,75	1,66	1,97	1,89	2,07
1,04	1,18	1,84	1,68	1,55	2,36
1,76	2,2	2,18	1,89	2,09	3,05
1,98	1,7	1,48	1,91	1,77	3,27
2,21	1,81	1,69	1,92	1,73	2,66
2,44	1,68	1,48	2,26	1,83	1,98
2,26	1,72	1,84	1,83	1,67	1,89
2,06	1,91	2,19	2,67	2,09	3,29
1,44	0,91	1,78	1,48	1,57	4,75
1,31	2,31	3,63	2,72	2,72	3,58
1,36	1,59	2,2	2,46	2,19	3,35
0,81	0,47	0,99	1,57	1,65	3,83
1,84	2,23	1,88	2,74	2,11	5,57
0,46	0,45	2,07	2,16	1,33	3,88
2,32	1,38	1,76	1,83	1,3	4,04
0,99	1,49	1,91	2,13	1,89	5,78
0,94	1,21	2,4	1,43	1,29	4,33
1,04	1,36	2,39	2,09	1,67	2,36
**1,5565**	**1,4955**	**1,943**	**2,0325**	**1,8155**	**3,5355**

Column289	Column290	Column291	Column292	Column293	Column294
A:418(SER)	A:419(PHE)	A:420(ARG)	A:421(ARG)	A:422(ALA)	A:423(PHE)
2,44	1,96	3,06	2,97	2,42	1,87
1,66	1,54	2,04	1,76	1,51	1,16
1,6	1,38	2,75	2,82	3,03	2,97
1,13	0,52	1,53	1,34	1	1,13
1,46	1,49	1,81	2,44	2,2	2,32
1,83	1,55	2,14	2,31	2,3	2,28
1,26	1,32	1,25	1,66	1,39	1,51
1,98	1,82	1,54	1,95	2,13	1,66
1,36	1,82	1,91	1,4	1,64	1,63
2,11	2,01	2,88	2,77	2,17	1,57
1,92	1,57	2,5	3,75	3,12	2,51
2,95	2,29	2,68	3,24	2,43	2,08
2,3	1,78	2,17	3,62	2,3	1,75
1,87	1,29	1,97	2,69	1,87	1,71
2,43	2,04	2,35	2,61	2,25	2,18
0,47	0,4	0,99	1,18	0,53	0,43
1,25	0,99	2,65	0,89	0,97	0,75
2,07	1,58	2,18	2,35	1,78	1,46
0,87	1,11	1,64	0,98	1,28	1,35
1,26	0,96	1,47	1,58	0,88	0,99
**1,711**	**1,471**	**2,0755**	**2,2155**	**1,86**	**1,6655**

Column295	Column296	Column297	Column298	Column299	Column300
A:424(THR)	A:425(LYS)	A:426(LEU)	A:427(LEU)	A:428(CYS)	A:429(PRO)
3,19	3,66	3,17	2,8	5,57	9,93
2,35	2,43	2,16	1,73	6,65	9,23
4,47	4,73	4,83	5,7	6,38	12,23
1,57	2,67	2,75	3,77	5,88	10,89
3,45	3,41	3,33	4,81	7,63	12,12
3,17	3,35	3,29	5,07	6,8	10,74
2,11	2,34	2,29	3,52	5,37	9,84
2,33	2,32	2,13	4,11	5,3	10,42
1,55	1,47	1,57	2,25	4,15	7,91
2,02	1,85	2,03	3,38	4,62	8,43
4	5,33	5,09	5,37	6,67	13
2,17	2,36	1,96	1,04	4,77	7,17
2,3	2,87	2,43	1,02	4,5	7,51
2,6	2,77	2,52	2,27	5,48	8,88
2,51	2,45	2,44	2,21	6,64	8,16
1,26	0,62	0,6	1,14	4,1	5,34
1,55	0,95	1,44	1,45	5,51	6,24
2,14	2,21	1,61	1,29	5,08	6,64
1,81	1,83	1,46	1,47	3,74	5,69
1,13	1,46	0,92	1,15	3,67	7,1
**2,384**	**2,554**	**2,401**	**2,7775**	**5,4255**	**8,8735**

Column301	Column302	Column303
A:430(GLN)	A:431(LYS)	A:432(LEU)
6,98	13,25	15,21
9,3	15,49	15,72
7,59	10,94	16,32
9,42	14,59	16,12
9,96	15,64	17,75
10,1	14,3	18,33
7,97	12,28	16,7
8,45	12,21	16,61
4,25	10,59	13,01
5,5	12,17	13,54
7,98	11,57	15,75
5,83	13,08	13,59
4,94	12,16	13,18
6,14	13,78	14,49
7,16	14,26	14,01
5,42	11,95	11,56
7,36	13,9	12,63
6,16	13,17	16,02
3,93	11,21	11,3
3,7	10,12	11,89
**6,907**	**12,833**	**14,6865**
